# The Curved Openspace Algorithm and a Spike-Latency Model for Sonar-Based Obstacle Avoidance

**DOI:** 10.3389/fnbot.2022.850013

**Published:** 2022-06-01

**Authors:** Chenxi Wen, Timothy K. Horiuchi

**Affiliations:** ^1^Department of Electrical and Computer Engineering, University of Maryland, College Park, MD, United States; ^2^Neuroscience and Cognitive Sciences Program, University of Maryland, College Park, MD, United States

**Keywords:** attention, winner-take-all, bat echolocation, neural model, spike latency, collision avoidance, robotics

## Abstract

The rapid control of a sonar-guided vehicle to pursue a goal while avoiding obstacles has been a persistent research topic for decades. Taking into account the limited field-of-view of practical sonar systems and vehicle kinematics, we propose a neural model for obstacle avoidance that maps the 2-D sensory space into a 1-D motor space and evaluates motor actions while combining obstacles and goal information. A two-stage winner-take-all (WTA) mechanism is used to select the final steering action. To avoid excessive scanning of the environment, an attentional system is proposed to control the directions of sonar pings for efficient, task-driven, sensory data collection. A mobile robot was used to test the proposed model navigating through a cluttered environment using a narrow field-of-view sonar system. We further propose a spiking neural model using spike-timing representations, a spike-latency memory, and a “race-to-first-spike” WTA circuit.

## Introduction

Traveling through an environment toward a goal without colliding with obstacles is one of many essential abilities for animals to survive. Animals are often able to detect obstacles using different types of sensors to quickly decide on the motions to avoid them. In addition, animals are often observed to orient their heads in different directions to gather sensory information needed for obstacle avoidance. In the world of robotics, there have historically been two extreme philosophical starting points in the approach to solving this problem: rigorous path planning assuming accurate and extensive sensing (Latombe, 2012) and fast reflexive behaviors based on minimal and unsophisticated sensing (Braitenberg, 1986). Clearly, there is an expansive world of algorithms lying between these two extremes. Path planning algorithms calculate routes between starting and goal points, requiring extensive knowledge of the environment and accurate localization. These are appropriate when a tremendous amount of relevant knowledge about the world is available and optimal paths are desired. In contrast, reflexive algorithms simply steer the creature away from obstacles upon detection with very little latency (Milde et al., 2017). Although reflexive behaviors are well-suited to a creature traveling quickly through an unknown or changing sparse environment, even mildly cluttered environments can produce inappropriate movements. Philosophically, obstacles should not determine the direction in which a creature should move, rather they should simply indicate where the creature should *not* go. The question is then, “given the information about multiple sensed obstacles and the target location, how do we combine them to select a good path?”

Echolocating bats are excellent examples of creatures that possess such a capability. They predominantly use ultrasonic echoes to perceive their surroundings and fly through dense forests in complete darkness with ease. During their hunt for flying insects or other prey, they can avoid obstacles while homing in on their prey at the same time. Big brown bats (*Eptesicus fuscus*), using sonar calls that last 2–3 ms, send out sonar pulses to detect obstacles at a rate of up to 90 Hz and have been shown to fly amongst obstacles at a flight speed of 2–5 m/s in indoor environments (Sändig et al., 2014). Big brown bats are also observed to turn their heads to ping in different directions to gather sensory information while flying in a field of obstacles (Surlykke et al., 2009). Understanding how biological systems can deftly transform the storm of sensory information into motor actions to pursue a goal while avoiding obstacles has long been a goal for engineers and neuroscientists.

Sonar has several advantages compared to other sensing domains (e.g., vision, LIDAR, or infrared sensors) commonly utilized in animals or robot systems. For example, sonar has the capability of penetrating smoke or fog where LIDAR, infrared sensors or cameras can struggle. Sonar also works in all light conditions, whereas it can often be difficult for LIDAR and infrared sensors to work in bright lighting, and cameras often struggle to work in complete darkness. Regardless of the sensing modality, once obstacles have been detected, there are many proposed approaches to this local obstacle-avoidance problem for a robot or a vehicle. One popular set of approaches is based on vector summation. In these approaches, obstacles create repulsive force fields and goals create attractive force fields. The summation of these forces steers the vehicle along a safe path without colliding with obstacles. Some specific implementations are APF (Artificial Potential Fields) (Khatib, 1986; Lyu and Yin, 2019; Rostami et al., 2019; Shin and Kim, 2021) and VFF (Vector Field Force) (Borenstein and Koren, 1989). These algorithms are effective and interesting in their computational simplicity and mathematical elegance. They have problems such as the vehicle being trapped in local minima, although recent modifications have been proposed (Rostami et al., 2019) to solve the local minima problem. As mentioned earlier, however, we believe that obstacles should not turn the vehicle in any particular direction but only indicate where it should not go.

Other approaches to navigation on a planar floor divide the surroundings of the vehicle into angular sectors and transform them into a polar histogram. In this histogram, the proximity of obstacles in each sector is represented and the next direction in which to steer is calculated based on the values of the histogram. These approaches include VFH (Vector Field Histogram) (Borenstein and Koren, 1991; Wu et al., 2020), its extension VFH+ (Vector Field Histogram Plus) (Ulrich and Borenstein, 1998) and the “Openspace” algorithm (Horiuchi, 2009). These approaches are good for local maneuvering but are centered in the sensory domain and do not generally consider vehicle kinematics. There are also velocity methods that map the Cartesian space into the velocity space that represents the linear and angular velocities of the vehicle, then calculate the next movement of the vehicle in the velocity space. These methods are suitable for differential or holonomic vehicles because a point in the velocity space corresponds to a velocity that is directly executable on the vehicle. The algorithm evaluates a range of possible velocities in the velocity space according to an objective function that includes criteria such as speed, distances of the obstacles, or goal direction. Some specific implementations include CVM (Curvature Velocity Method) (Simmons, 1996; Molinos et al., 2014), DWA (Dynamic Window Approach) (Fox et al., 1997), and its recent extension DW4DO (Dynamic Window for Dynamic Obstacles) (Molinos et al., 2019).

There are collision avoidance algorithms that rely on deliberate planning (Aggarwal and Kumar, 2020; Yasin et al., 2020). In these algorithms, an optimal or near-optimal path with collision-free routes is calculated based on an environmental map that the vehicle senses and updates. These algorithms typically assume extensive, accurate maps over which long trajectories are tested sequentially, requiring both significant computational resources and memory as well as fast, accurate sensing. To address the high computational complexity of these algorithms, several optimization methods have been developed. In Pérez-Carabaza et al. (2019), a minimal time search algorithm with ant colony optimization is used to calculate the optimal path under communication-related constraints. The algorithm in Bry and Roy (2011) incrementally constructs a graph of trajectories while efficiently searching over candidate paths, resulting in a search tree in belief space that converges to the optimal path. Using this algorithm, aggressive flight of a fixed-wing air vehicle in an unstructured 3D environment was demonstrated (Bry et al., 2012). Maintaining and updating a metrically correct spatial map, however, is difficult to implement in a biologically plausible neural system.

**Figure 1:**
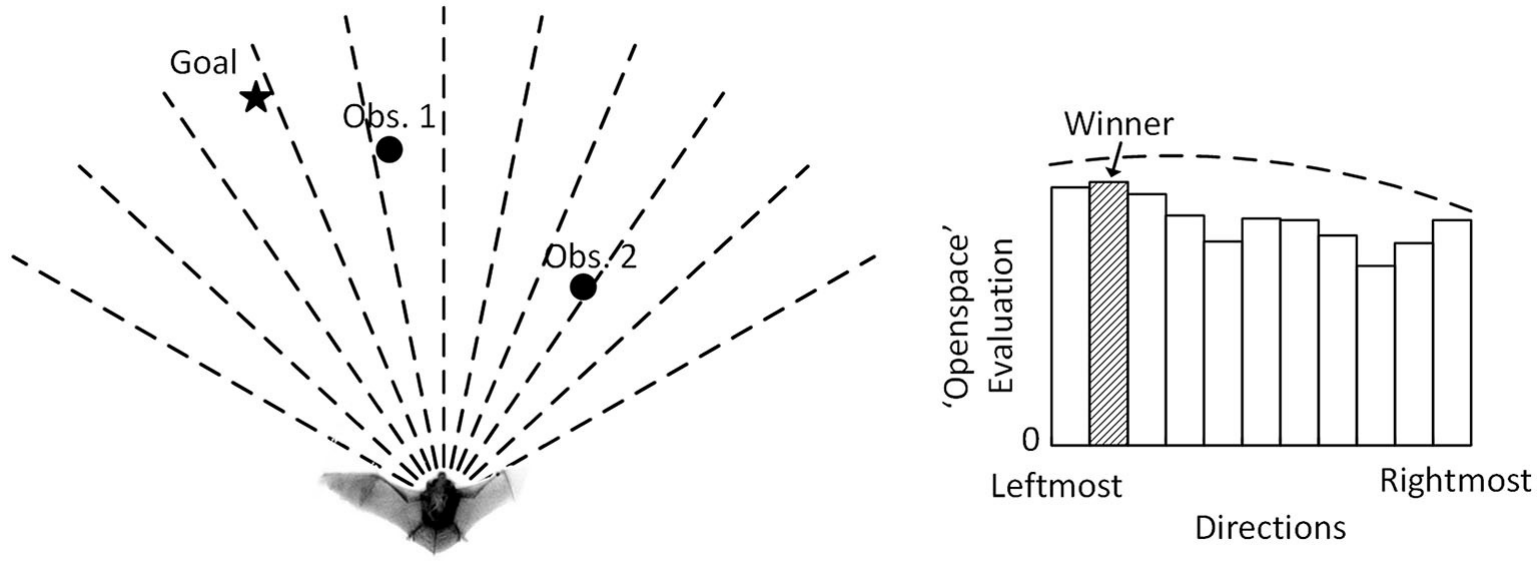
The Openspace algorithm of Horiuchi (2009). (Left) An echolocating bat that is attempting to fly to the goal (filled star) while avoiding two obstacles (filled circle). (Right) The evaluation pattern consists of a constant plus a wide low-amplitude Gaussian (Goal input) with two dips created by the suppression from the two obstacles. A winner-take-all (WTA) function selects the direction with the highest evaluation (filled bin). The dashed line indicates the default evaluation with no obstacle present.

**Figure 2:**
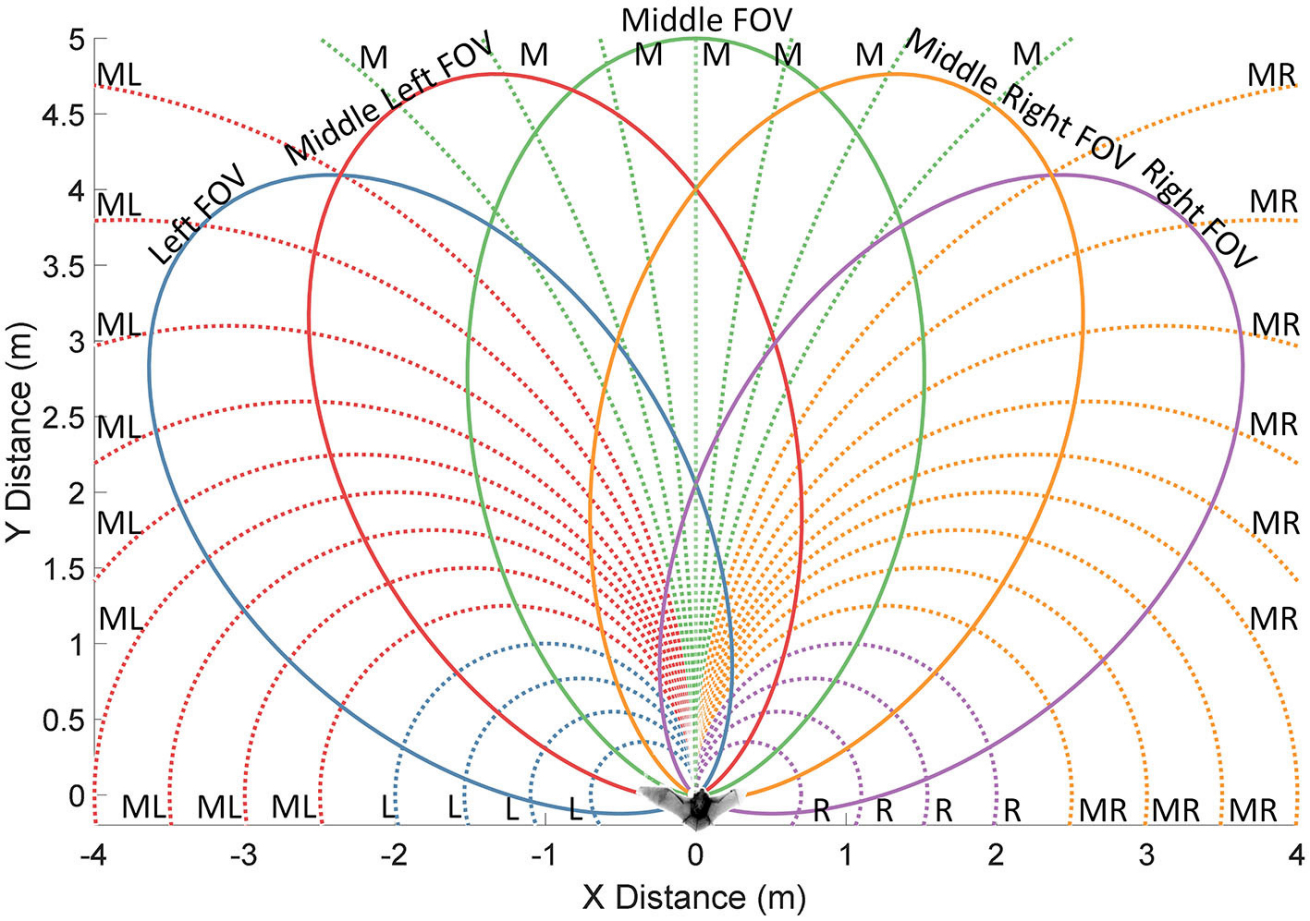
An illustration of motor choices, sonar field of view (FOV) and groupings of the motor choices. Selecting different motor choices results in paths as circular arcs with different radii (dotted lines). The sonar FOV (solid lines) is ellipsoidal and the bat can turn its head to ping in different directions. Five ping directions and 33 motor choices are shown. The colors along with the letters at the end of the paths indicate which groups (left, middle-left, middle, middle-right or right) the motor choices belong to.

The Openspace algorithm proposed in Horiuchi (2009) provides a neuromorphic VLSI implementation of the sensory-oriented histogram approach with a latency-based, spiking neural network, giving insights into how a biological system might implement sonar-based navigation. The Openspace algorithm seeks to find the most desirable straight-line direction of travel to avoid obstacles (Figure 1) while a bat is traveling on a 2-D plane. It divides the area in front into a number of steering directions, evaluates their desirability, and selects the winning direction with the maximum evaluation. It combines different inputs (a goal direction and detected obstacles) into a decision function to determine the steering decision. To physically travel precisely in the selected direction, however, a bat must be capable of making extremely sharp turns. In practice, a flying bat can only rotate gradually to the desired direction. This produces an overshoot that will require ongoing corrections that lead to a mismatch between the selected path and the actual path the bat flies on. In contrast, our proposed algorithm, the Curved Openspace Algorithm, projects the sensory-based obstacle data into “motor coordinates” before comparing motor choices similar to the velocity approaches (Simmons, 1996; Fox et al., 1997; Molinos et al., 2014). For a flying bat, this could mean selecting different turning radii (i.e., circular trajectories) as illustrated in Figure 2.

Another limitation of the original Openspace algorithm is the assumption that the bat has a wide field-of-view (FOV) that covers all directions with the same effective range, thus localizing all obstacles in front of the animal with a single sonar ping. A practical sonar system, however, has a limited field-of-view whose detection range is angle-dependent due to the beam patterns of both the sonar transmitter and the receivers, resulting in an ellipsoidal FOV (Figure 2). For big brown bats, the half-power beamwidth (the angular width of the beam pattern at the 3 dB cutoff points) of their emitted ultrasonic signal at 35–40 kHz is ~56–80 degrees (Ghose and Moss, 2003; Gaudette et al., 2014). In the simulations shown here, a Gaussian-shaped FOV with a standard deviation of 30 degrees was used, resulting in a half-maximum width of 70.7 degrees. This limited FOV means that a bat will need to sequentially probe different directions to make good steering choices, which is time-consuming and leads to choices based on old data. Although engineered systems frequently employ continuous side-to-side scanning, this is *not* observed in echolocating bats flying through a field of obstacles. For a well-defined task like steering toward a goal, when a clear path toward the goal is detected, no scanning is needed. In this paper, we propose a novel neural model to find a collision-free path using attentional search to guide the movement of sensors. The selected paths correspond to the natural curvature of bat flight.

The Curved Openspace neural model, like the original Openspace algorithm, combines echoes from a 2-D (azimuth and range) sonar to create an evaluation for each of the different motor actions under consideration. Due to the limited field-of-view of the sonar, a memory is needed to hold these evaluation values as the sonar interrogates different directions. To avoid excessive scanning, we introduce an attentional system that integrates information about a goal direction and stored action evaluations to determine where (or if) to turn the sonar for the next ping. Instead of constructing and updating an expensive 2-D map about all the obstacles in the memory of a bat, we can build a significantly simpler system by collapsing the 2-D sensory map into a 1-D evaluation memory among different motor action choices. Each motor action represents an arc of travel through the environment and its evaluation represents how risky it is. Having the evaluation memory to combine sensory inputs across head turns enables an action selection layer to select the most desirable path using a winner-take-all (WTA) function. Unlike the immediate motor response of the original Openspace algorithm with each sonar ping, the action selection (WTA) layer only provides preliminary decisions that are further processed before making a final decision. It should be noted that although the proposed model is described with a sonar system, it can work with any type of input sensor without significant changes to the structure of the model.

We describe the neural model in detail in Section The Curved Openspace Neural Model and we propose a spiking neural model in section spike-latency neural model that takes advantage of the inherent time representation that originates in the sonar. In section experiment results, we show the simulation results of the proposed model in a dense forest where we collect statistics and investigate the effects of different features and parameters, and we show the simulation of the spiking neural model with comparable performance. We also validated the use of the model on an inexpensive mobile robot with a limited-FOV sonar in a “pipe forest.”

## The Curved Openspace Neural Model

The purpose of the Curved Openspace model is to generate a series of motor actions driven by a realistic sonar system to guide a bat-like agent to a goal location while avoiding obstacles on a 2-D plane.

**Figure 3:**
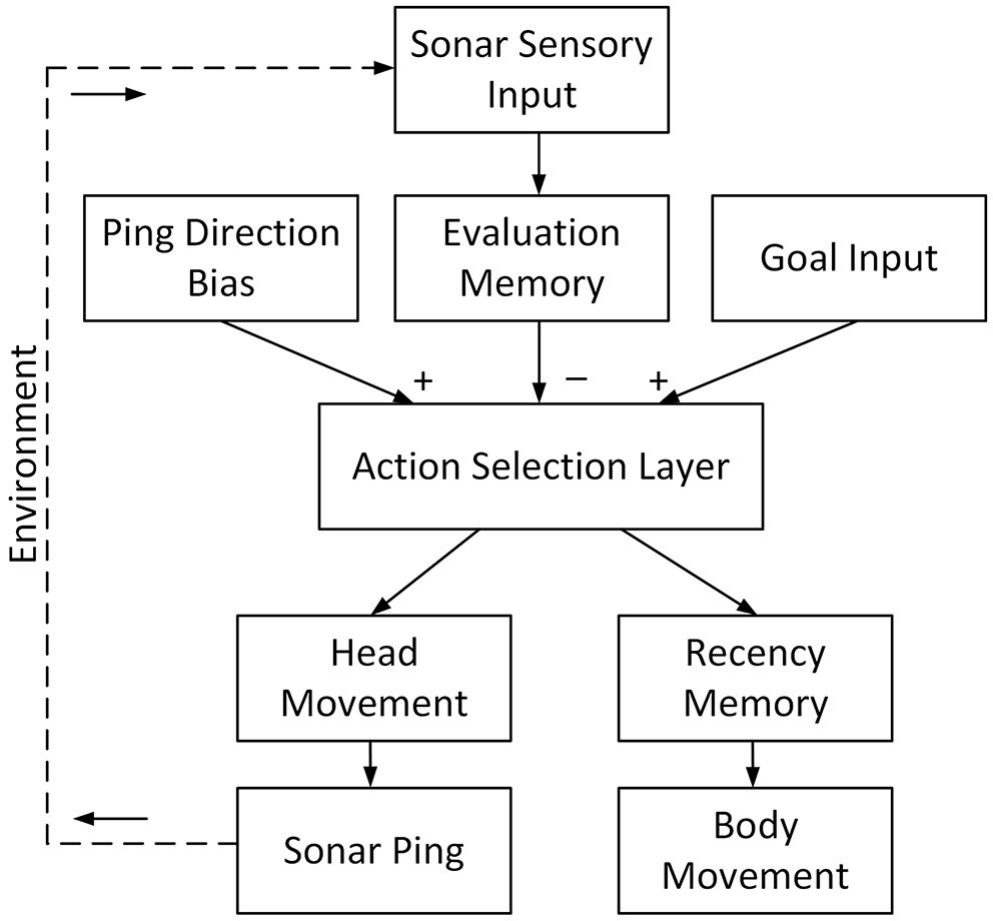
The system structure of the proposed model. After a sonar ping, the sensory input updates the evaluation memory. The evaluation memory, representing the risk of each path, inhibits the action selection layer. The direction of the current ping excites the action selection layer to encourage selecting paths with the most recent information. The goal input represents the direction of the goal and excites the action selection layer. The action selection layer then selects the winning path with a winner-take-all (WTA) function and the winning path drives the head movement of the bat to direct its next ping in the associated direction. In the meantime, the recency memory checks if the sensory information on the winning path is recent. If so, the winning path drives the bat to turn its body and fly on it.

The bat first localizes obstacles inside its field of view and sends the information to the evaluation memory (Figure 3) via a mapping that reflects different motor actions. The evaluation memory then combines new information with previous results to provide an evaluation of the *risk* along the trajectories of different motor choices. It suppresses the action selection layer with a Gaussian-shaped projection of inhibition. The action selection layer is excited by the goal input and uses a WTA function to calculate the most desirable motor choice. To fine-tune the action selection, other inputs, such as winner hysteresis and ping-direction bias, are added. Following the WTA, a “recency” condition is used to decide if the selection of the winning motor action (i.e., path) was based on recent information. If so, the bat is confident that the path is clear and executes the winning motor action. If the sonar has not sampled the winning motor action's direction recently, the bat will then ping in that direction, but will not execute the winning motor choice until it has.

In this model, it is assumed that the bat has a limited selection of motor choices, which includes a straight path and several curved paths (Figure 2). The straight path represents moving straight forward, and the curved paths are circular arcs with different radii consistent with the bat flying with a fixed turning rate. The bat is limited to ping in five fixed directions with respect to the body orientation (Figure 2). Depending on the portion of a path that falls into the field of view of a ping direction, individual paths are assigned to one of five view groups so that each path is associated with a single ping direction (Figure 2). We will refer to this neural model as the “analog” model when comparing it to a different implementation in Section Spike-Latency Neural Model.

### Zone of Collision

**Figure 4:**
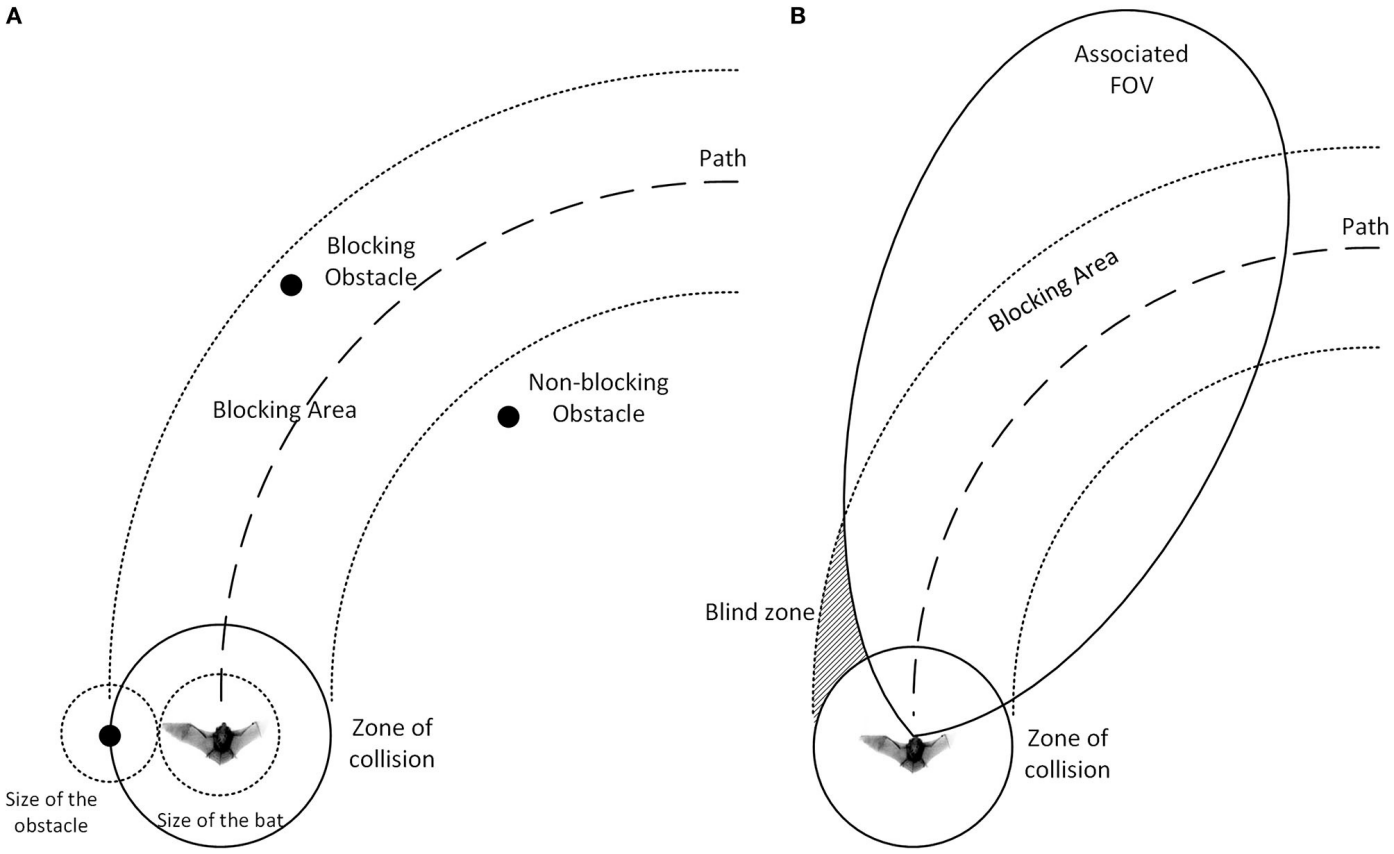
**(A)** The definition of the zone of collision. The zone of collision is a disk around the bat with a radius equal to the sum of the radius of the bat and the radius of the obstacle. With this definition, we can treat obstacles as points and the bat as a disk. The area where the obstacles are blocking the path (dashed line) is defined as the blocking area (dotted line). **(B)** An example of the “blind zone” where the associated FOV cannot cover. Obstacles can disappear from the FOV and move into the blind zone while still blocking the path. Taking the maximum value between the memory and the most recent evaluation can help the bat remember the existence of the obstacle and steer away accordingly.

We define the radius of the bat to be its maximum wingspan and we define the zone of collision to be a disk around the center of the bat (Figure 4A). The bat is considered to collide with an obstacle if the obstacle passes into the zone of collision. If the minimum distance between an obstacle and a path is smaller than the radius of the zone of collision, the obstacle is defined to be a blocking obstacle since traveling on this path will eventually lead to a collision. In this paper, the bat is modeled without flapping wings and cannot perform agile maneuvers with wings to avoid contact with obstacles as might occur in the real world.

### Speed Control

The traveling speed of the bat is controlled according to a “speed profile” that assigns a certain constant speed to each trajectory. The speed along a path *p* is selected as


(1) $$\begin{array}{r} v_{p}=V_{\text{MAX}}\cdot \gamma_{p} \end{array}$$

where *V*_MAX_ is a constant maximum speed and γ is the speed profile that varies from 0.0 to 1.0 for each path. Because the sonar is only able to observe different distances along each trajectory, the speed is adapted to normalize the risk for comparison across a fixed amount of time (i.e., the time between sonar pings). The straight, middle path has the longest observability and thus supports the highest speed. Hence, the speed profile can be calculated as


(2) $$\begin{array}{r} \gamma_{p}=R_{p}/R_{\text{MAX}} \end{array}$$

where *R*_*p*_ is the length of path *p* in its associated FOV and *R*_MAX_ is the maximum distance that the sonar system can detect. The speed profile aims to give the bat a constant reaction time on each path between a blocking obstacle becoming detectable and colliding with the bat. Intuitively, if the bat can detect obstacles further into the future on a path, it should be more comfortable with flying at a higher speed on that path. Sharp turns, however, usually require the bat to fly at a lower speed since the FOVs to the side only cover a short length of the path and any detectable obstacles in the blocking area are already close to the bat (Figure 2). Implementing the “speed profile” for speed control reduces the chance of collision during sharp turns.

### Evaluation Memory

The 1-D evaluation memory represents the collision risk of different paths based on new and old sensory information. Each bin of the evaluation memory corresponds to a motor action choice. The analog value of the memory represents “risk” that combines the immediacy of avoidance and the concept of the growing uncertainty of the locations of blocking obstacles due to the movement of the bat. The *immediacy* of avoiding a blocking obstacle *j* on a path *p* is represented as a function (*f*).


(3) $$\begin{array}{r} f_{p}\left(j\right)=E_{\text{MAX}}\cdot \left[1-\frac{r_{p}\left(j\right)-R_{C}}{R_{\text{MAX}}\cdot \gamma_{p}}\right] \end{array}$$

where *E*_MAX_ is a constant representing the maximum value of *f*_*p*_. *r*_*p*_(*j*) is the distance from the bat to a blocking obstacle *j* along path *p* approximating the obstacle to be on the closest spot on path *p*. *R*_*C*_ is the radius of the zone of collision, and *r*_*p*_(*j*)−*R*_*C*_ represents the distance that the bat can travel along path *p* before colliding with obstacle *j*. *R*_MAX_ is the maximum distance that the sonar system can detect and γ_*p*_ is the speed profile for path *p*. According to Equation 1 and the relationship that *r*_*p*_(*j*) ≤ *R*_*p*_, the second term in Equation 3 is smaller than 1, resulting in a positive *f*_*p*_(*j*). The second term indicates how long the bat can travel on path *p* before colliding with a blocking obstacle *j*. A closer blocking obstacle on a path with a higher speed profile will result in a higher immediacy *f*_*p*_.

To evaluate the risk of a given motor action choice *p* (a path), we use the following equation:


(4) $$\begin{array}{l} {\text{E}}_{p}(t)=\text{max}[E_{p}(t-\Delta t)-\beta (t-\Delta t)\cdot I_{d}-\alpha (p)\cdot I_{inh},\\ \text{New Previous Passive Directional}\\ \text{value value decay inhibition}\\ \;min\left(\sum_{\left(j\epsilon B_{p}\right)}f_{p}(j),E_{\text{MAX}}\right)]\\ \text{ Summation of the immediacy}\\ \text{ of avoidance (saturating)} \end{array}$$

where


$$\begin{array}{l} \alpha (p)\;=\{\begin{array}{cc} 1, & if\;path\;p\;is\;associated\;with\;the\;direction\;of\;the\\ & current\;ping\\ 0, & otherwise \end{array}\\ \beta (t)\;=\{\begin{array}{cc} 0, & decay\;is\;halted\\ 1, & otherwise \end{array} \end{array}$$

where *E*_*p*_(*t*) is the evaluated risk for a path *p*, and the new risk is the maximum between a decayed memory and a saturating summation of the immediacy of avoiding blocking obstacles. β(*t*) represents a passive decay whose strength *I*_*d*_ is a constant. The passive decay represents the increasing uncertainty of the location of obstacles with time and is updated at every time step except when the bat enters a “scanning mode” and halts the passive decay. α(*p*) is a binary value that becomes 1 when the bat sends a ping and path *p* is associated with the direction of the ping. It represents a directional inhibition where the strength of the inhibition *I*_*inh*_ is a constant. B_p_ is a set of the obstacles blocking path *p* and it defaults to an empty set when the bat does not send out a sonar ping at time t, resulting in a summation term of 0. This summation term represents “risk,” where obstacles with higher immediacy of avoidance pose a higher risk and this risk accumulates with every blocking obstacle along the path. The risk is then saturated (using the minimum function) if it gets higher than the maximum evaluation value *E*_MAX_. The final evaluation value after the update is the larger of the decayed old value and the new risk value computed from the objects currently being sensed. Because the FOV that a path is associated with cannot cover the whole blocking area of the path, a blocking obstacle can disappear from the current field of view while still being a threat (Figure 4B). The max function is thus used as a more conservative assessment of risk between the memory and what the sensory system is detecting. Essentially, the evaluation memory is updated with new information if a sonar ping is sent at time t, and the memory is kept with or without decay (depending on β) when there is no sonar ping.

The evaluation memory allows the bat to combine path evaluations gathered through several pings in different directions. Since the bat is still moving while gathering information, the stored memories can become outdated. By default, the values of the evaluation memory decay quickly over time to represent an increase in the ambiguity of obstacles (constant “passive decay”). When the bat changes its ping direction, the decay is temporarily halted until a motor choice has been selected and executed. In this “attentional search mode,” the bat pings rapidly in the different directions of interest to minimize the distance traveled between pings and the loss of accuracy in the memory due to movement.

When a sonar ping occurs, the evaluation memories of the paths associated with the current ping direction are also inhibited. This “directional inhibition” is shown as the term α(*p*)·*I*_*inh*_ in Equation 4. Since the maximum function will keep the previous evaluation memory if the bat did not detect any obstacles blocking the path, the path might be more open than what the memory suggests. The directional inhibition aims to reduce the risk of a path in this scenario.

### Action Selection Layer

In this model, collision avoidance is viewed as an attentional search for good paths, combining parallel search within the field of view of a single ping, but serial search across head movements (Itti and Koch, 2000). The action selection layer combines the collision risk calculation from the evaluation memory with goal information and other biasing signals to create a desirability value for each path (i.e., motor) choice. Having no obstacle on a path will give that path a low risk value, resulting in a high desirability in the WTA layer. Calculation of the desirability of each path occurs after every sonar ping and can be described by


(6) $$\begin{array}{l} \begin{array}{l} D_{p}=D_{0}(p)+G\cdot e^{\frac{-{\left(p-p_{g}\right)}^{2}}{\sigma_{g}^{2}}}+P\cdot \alpha (p)+H\cdot e^{\frac{-{\left(p-p_{h}\right)}^{2}}{\sigma_{h}^{2}}}\\ \;\begin{array}{l} \text{Constant Goal Ping Direction Hysteresis}\\ \text{Bias input Bias}\\ -W\cdot \sum_{p_{mem}=1}^{N}E_{p_{mem}}\cdot e^{\frac{-{\left(p-p_{mem}\;\right)}^{2}}{\sigma_{m}^{2}}}\\ \text{Inhibition from}\\ \begin{array}{c} \text{evaluation memory} \end{array} \end{array} \end{array} \end{array}$$

The first term *D*_0_(*p*) is a positive constant bias that represents default desirability. This term allows the evaluation to remain positive even while being reduced by other terms. Besides, each path could have a different bias term to incorporate additional information about the desirability of individual paths due to actuation limits or energy considerations. This was used in the simulations described in section experiment results to discourage sharp left or right turns. The coefficient *G* is the amplitude of an additive Gaussian term with its center at *p*_*g*_ and a standard deviation of σ_*g*_. This term provides a bias toward some motor actions over others due to externally provided information about a goal location. The magnitude of excitation from the goal input is much weaker in comparison to the inhibition from the evaluation memory since the goal input only serves as a bias toward the goal when different motor actions have similar risks levels. The third term is an excitation term from the current ping direction that biases the bat to select the path with the most recent information when several paths have similar risks. α(*p*) is the binary value defined in Equation 5 and *P* is the amplitude of the excitation. The coefficient *H* is the amplitude of a Gaussian term that produces hysteretic behavior, where *p*_*h*_ is the path that the bat is currently traveling on and σ_*h*_ is a constant that controls the width of the Gaussian. This term prevents the bat from changing paths erratically due to noise in the measurements or occupancy calculations. The index *p*_*mem*_ refers to the bins of the evaluation memory that suppress the desirability with a subtractive Gaussian term scaled with their values *E*_*p*_*mem*__ and an inhibition weight of *W*. The width of the suppression is controlled by σ_*m*_ that is kept constant. After the evaluation, the action selection layer selects the path with the maximum desirability for head and body control.

### Motor Control

The action selection layer determines the direction in which the head should be pointed (i.e., in the direction of the winning path). If a head movement is needed, it will be performed. There are two scenarios in which a path is selected. The first scenario is when the path has a low risk value after the bat has pinged in its direction. In this case, the bat will likely travel along this path. The second scenario is that the bat has not pinged in the direction of the winning path, and its desirability is high because the default risk value in the evaluation memory is low. In this situation, pinging in an unknown direction can help the bat explore possible open paths.

**Figure 5:**
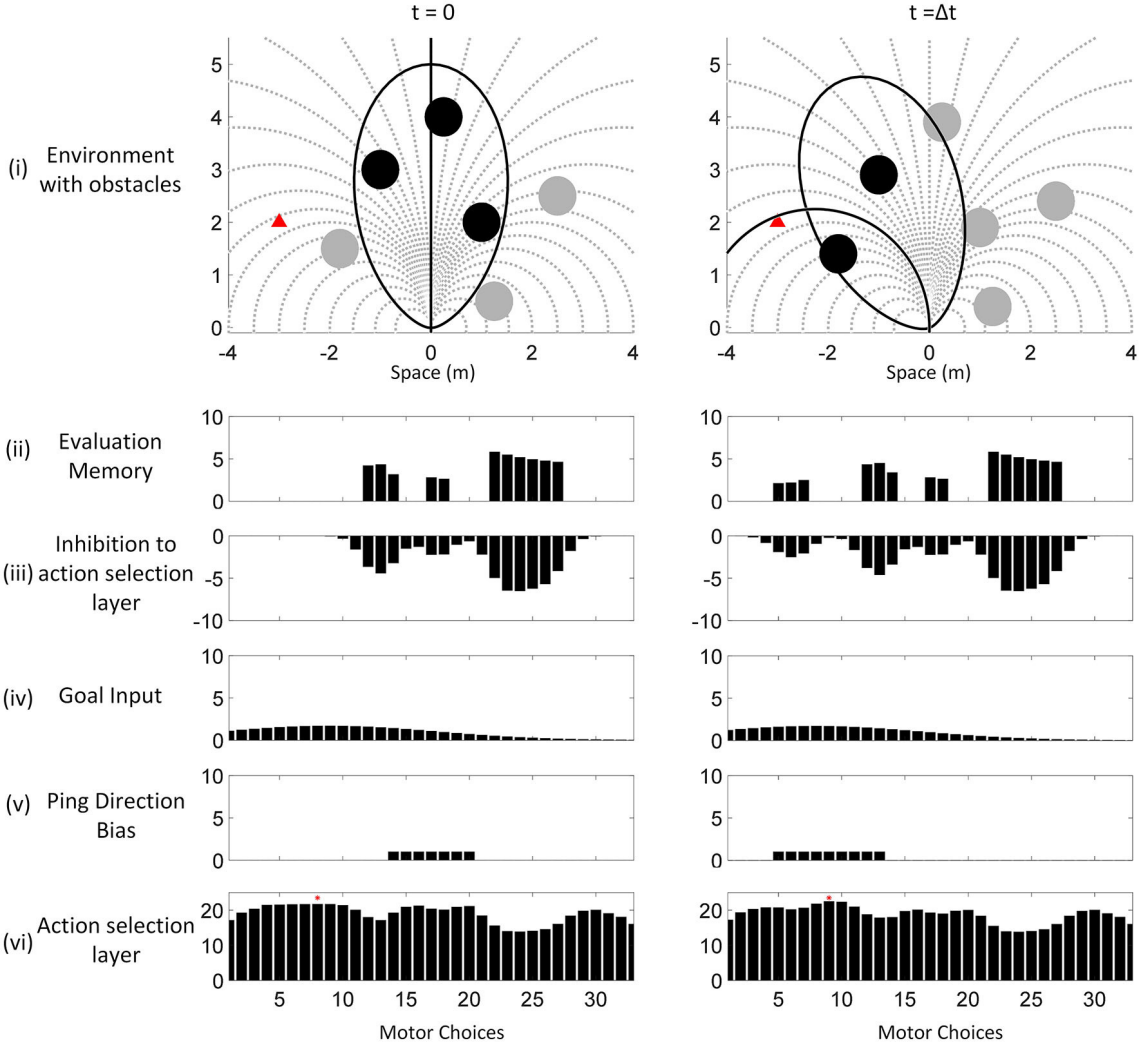
An example of the proposed neural model from sensory input to motor action selection. The environment, evaluation memory, values of the action selection layer, ping direction and motor actions in two timesteps from the simulation are shown. A bat was flying in a field with 6 obstacles (filled circle) and a desired destination (filled triangle in red). The circles of the obstacles are drawn with the size of the zone of collision to show the paths that they are blocking. The bat has a limited selection of motor choices (dotted line) and each of the motor actions corresponds to a bin in the bar graphs below. At *t* = 0, it is assumed that the bat arrived in the environment (i) with no prior information. It is also assumed that it was flying along a straight path (solid straight line) and it directed its first sonar ping to the front (ellipsoidal FOV shown in solid line). With the first ping at *t* = 0, it detected three obstacles (filled circle in black) and updated its evaluation memory (ii). The evaluation memory units suppressed the action selection layer with Gaussian projection of inhibition (iii) while the goal input imposed a wide Gaussian excitation (iv). Because the bat sent out its sonar ping toward the front, the associated motor actions received excitations as shown in the ping direction bias (v). The action selection layer (vi) selected the winning path (indicated with a red dot on top of the bar), which is gated by the recency memory (not shown). Because the winning path is associated with the middle-left ping direction and the bat had not pinged in that direction recently, it turned its head to send the next sonar ping to the middle-left, did not execute the winning path and entered the “scanning mode” that halted the passive decay on the evaluation memory. At the next timestep *t* = Δt, the bat pinged to the middle left direction and updated the evaluation memory. The goal bias stayed the same while the ping direction bias changed to excite the paths associated with the middle left direction. The action selection layer combined the information in the same way and selected a path as the winner. Since the winning path belongs to the middle-left group and the bat just pinged in the same direction, the bat executed the winning path (solid line), exited the “scanning mode” and allowed the evaluation memory to decay. Notice that with the proposed model, the bat only pinged in the directions of interest to find the most desirable path and did not need to do a full scan.

For the bat to fly along a particular trajectory (i.e., *execute* a motor action,) the path must be chosen by the action selection layer ***and*** the sonar data evaluating that path must be fresh (a.k.a., “recent”). If the selected path is based on old data, a head movement (and a ping) is generated to obtain new data. Once both criteria are satisfied, the output of the action selection layer is allowed to change the bat's trajectory. At the same time, the bat exits “attentional search mode” and begins allowing its evaluation memory to decay. To keep track of data recency, each of the five ping directions has a countdown timer called recency memory that resets to a high value after a ping in its assigned direction. The recency memory of a ping direction must exceed a certain threshold for the bat to select paths associated with that direction. Note that the bat continues to travel along its prior trajectory until a new path (motor action) is selected for execution. An example of the behavior of the proposed model is shown in Figure 5.

When the desirability of the winning path is lower than a certain threshold (i.e., no acceptable paths were detected), an “emergency” is declared and the bat will do a sharp 180-degree turn and travel in the opposite direction to the path it was traveling on before the turn. It will also direct its next sonar ping to the associated direction of the traveling path. This emergency “turnaround” maneuver is vital in the simulation for the bat to escape from the scenario where all paths are blocked by obstacles (a.k.a., a trap). Bats in the real world often perform this by using the vertical dimension to abruptly fly straight up, turn around and fly back down in the opposite direction (similar to the “hammerhead” maneuver in airplanes).

The Curved Openspace model was simulated in a dense forest where we investigate the effects of different parameters in section simulation of the analog model. The model was also implemented on a mobile robot with a car-like steering mechanism in section robot implementation. We show that the robot is able to travel in a dense forest of plastic pipes without collision using a narrow-FOV sonar system mounted on a head-turning servo motor.

## Spike-Latency Neural Model

**Figure 6:**
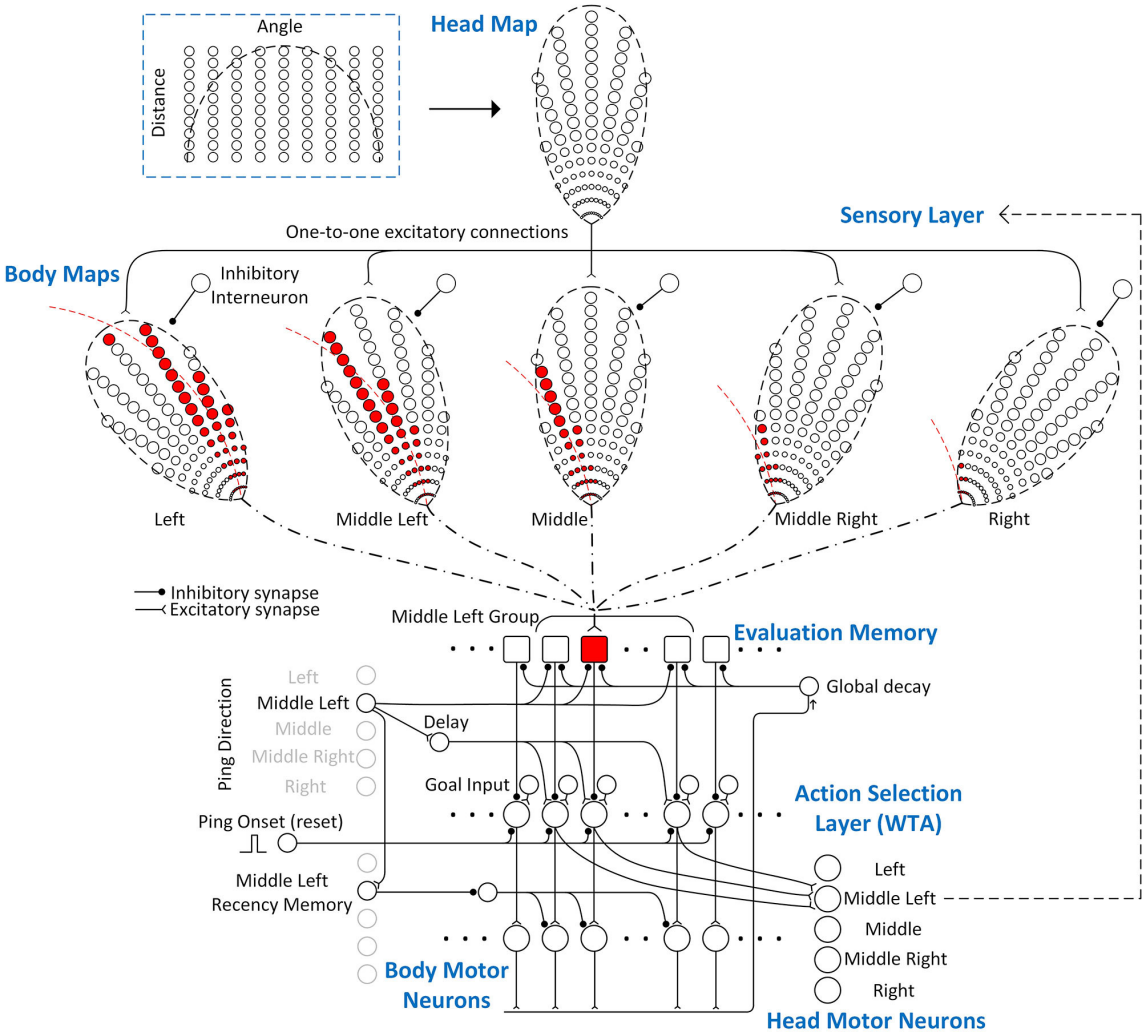
The spiking neural model of the Curved Openspace model. It mainly consists of four layers: a 2-D sensory layer encoding the locations of obstacles, a memory layer storing the “risk” of different paths, an action selection layer that uses a “race-to-first-spike” WTA mechanism to select the winner and a motor layer that controls the body and head movements. Only a portion of the connections in the group of middle-left ping direction are shown in this example for clarity. The sensory layer consists of a 2-D sensory map in head frame (head map) and five sensory maps in body frame (body maps). The elliptical dashed lines around the maps represent the field of view of the bat and the circles represent sensory neurons which fire when obstacles are detected in their locations. The number of neurons in each map is reduced for clearer illustration. Each body map has an inhibitory neuron that inhibits all the neurons in the map when it fires a spike. The neurons in the head map strongly excite the neurons in the same positions in each body map but only one of the body maps will not be inhibited by the inhibitory interneuron after a sonar ping, depending on the ping direction. The neurons in the body maps make fixed excitatory connections (dash-dotted line) to the evaluation memory units if the represented obstacles in their positions are blocking the paths. One example of the connections between the body maps and an evaluation memory unit is shown in red.

Although the analog model presented above can be implemented using large populations of spiking neurons to simulate (noisy) analog signal representations, spike-timing-based signal representations often suggest very different neural implementations with far fewer neurons. In this section, a spiking neural network model using spike-timing to represent signal values is described. As shown in Figure 6, its structure consists of four main layers: a sensory layer that encodes the 2-D locations of obstacles, a memory layer that integrates and stores the sensory information associated with different paths (“Evaluation Memory”), an “Action Selection” layer that uses a “race-to-first-spike” winner-take-all (WTA) mechanism to select a path, and a “Motor” layer that implements the body steering decision and head movements. Inputs to the spiking neural model also include ping directions (head direction), a global reset signal, goal direction input, and a ping onset signal.

The time at which a neuron fires a spike following an outgoing echolocation pulse is affected by many variables. Echolocation is foundationally based on the time-of-flight of sound to determine the distance to objects, with the closest objects generating echo signals first, producing a natural temporal coding scheme. Additionally, echoes from a given object are louder if it is closer. Interestingly, neurons commonly exhibit shorter latency responses to larger magnitude signals. In this model, following each sonar ping, sensory neurons will fire spikes with latencies that reflect the immediacy of avoiding any detected obstacles. The evaluation memory units integrate the sensory information on different paths and store the integrated information as spike latencies using a delay line and an array of latency memory units, which will be described in detail in Section Evaluation Memory. Because the stored signals are spike latencies, during readout, the evaluation memory units must be able to re-generate output spikes with the same latencies without sensory input. The evaluation memory sends out spikes with the stored latencies to the action selection layer, where the net desirability of different paths is compared, and a “winner” is selected. The neuron that fires the first spike in the action selection layer has the highest desirability and inhibits all other neurons in the same layer to prevent them from firing. The spike from the winning neuron is then sent to the motor neurons that will orient the sonar head for the appropriate ping direction (head motor neurons). The spike is also sent to the body motor neurons that produce the turn rate needed for the corresponding path. The body motor neurons, however, will only be activated if the recency memory of the associated ping direction is active. The gating from the recency memory is implemented with a disinhibition mechanism. The following sections describe each layer in detail.

### Sensory Layer

In this neural implementation, the sensory layer (Figure 6) uses a 2-D head map to represent the locations of obstacles and converts the information from the head reference frame to the body reference frame with several body maps. The neurons in the head map make strong one-to-one excitatory connections to the neurons with the same positions in all the body maps. The number of body maps is the same as the number of possible ping (i.e., head) directions and there is an inhibitory interneuron for each body map that strongly inhibits all the neurons in the map when it fires a spike. In this model, we define the spike latency as the time between the outgoing sonar ping and the first spike from a neuron. When the bat pings and detects an obstacle, the neuron at the corresponding location in the head map fires a spike with a latency close to the latency of the echo. Depending on the direction of the sonar ping, only one body map will be selected as active by inhibiting all other body maps using the inhibitory interneurons. A spike from the head map excites the corresponding neuron in the active body map and causes it to fire a spike immediately. The spikes from the active body map represent obstacles at certain locations in the bat's body frame. Because the Curved Openspace model uses the distance along a curved path (not the radial distance which is represented by the echo delay) to evaluate the risk value (Equation 3), the latencies of the spikes from the active body map are adjusted by adding extra latencies before the spikes are sent to the evaluation memory. Whether or not an evaluation memory unit takes synaptic inputs from neurons in the body maps is determined by whether obstacles in their positions are blocking the path that the evaluation memory unit represents. If they are blocking the path, the spikes from the corresponding neurons will be sent to the integrating neuron in the evaluation memory, which will be described in detail in section evaluation memory. The synaptic connections between the sensory layer and the evaluation memory are fixed if the paths are fixed.

The Openspace algorithm in Horiuchi (2009) made clever use of the natural latency of echoes as a representation of the straight-line (i.e., radial) distance to obstacles. In the Curved Openspace model, however, the immediacy of avoidance uses the distance along a curved path (Equation 3). As described earlier, to adjust the spike latency to reflect the immediacy correctly, each neuron in the body map connects to a delay neuron (not shown in Figure 6) that adds a *constant* latency specific to each position before connecting to the evaluation memory. The added latency for a connection between a sensory neuron *j* to the evaluation memory of path *p* can be calculated as


(7) $$\begin{array}{r} \Delta t\left(j,p\right)=T_{\text{MAX}}\cdot \frac{r_{p}\left(j\right)-R_{C}}{R_{\text{MAX}}\cdot \gamma_{p}}-t_{echo}\left(j\right)+T_{C} \end{array}$$

where Δ*t*(*j, p*) is the added latency and *t*_*echo*_(*j*) is the echo latency from an obstacle represented by neuron *j*. *t*_*echo*_(*j*) is constant for each sensory neuron since each neuron represents a fixed location on the body map. *T*_MAX_ is the echo delay from an obstacle at the maximum sensing distance *R*_MAX_ and is the maximum echo delay the sonar system can receive. The term $\frac{\left(r_{p}\left(j\right)-R_{C}\right)}{\left(R_{\text{MAX}}\cdot \gamma_{p}\right)}$ is the same term used in the immediacy function (Equation 3) that represents the time before the bat collides with an obstacle at the location of neuron *j*. *r*_*p*_(*j*) is the distance from the bat to a blocking obstacle *j* along path *p*, *R*_*C*_ is the radius of the zone of collision, *R*_MAX_ is the maximum distance that the sonar system can detect and γ_*p*_ is the speed profile for path *p*. The term has a value from 0 to 1, which makes the first term in Equation 7 have a value between 0 and *T*_MAX_. *T*_*C*_ is a small and constant delay to keep Δ*t*(*j, p*) positive. With the added latency, the spike latency that arrives at the evaluation memory of path *p* from sensory neuron *j* (if an obstacle is sensed) can be written as


(8) $$\begin{array}{r} t_{\text{spk}}\left(j,p\right)=\Delta t\left(j,p\right)+t_{\text{echo}}\left(j\right)=T_{\text{MAX}}\cdot \frac{r_{p}\left(j\right)-R_{C}}{R_{\text{MAX}}\cdot \gamma_{p}}+T_{C} \end{array}$$

A closer blocking obstacle on a path with a higher speed profile will result in a shorter spike latency, indicating a higher immediacy of avoidance.

### Evaluation Memory

**Figure 7:**
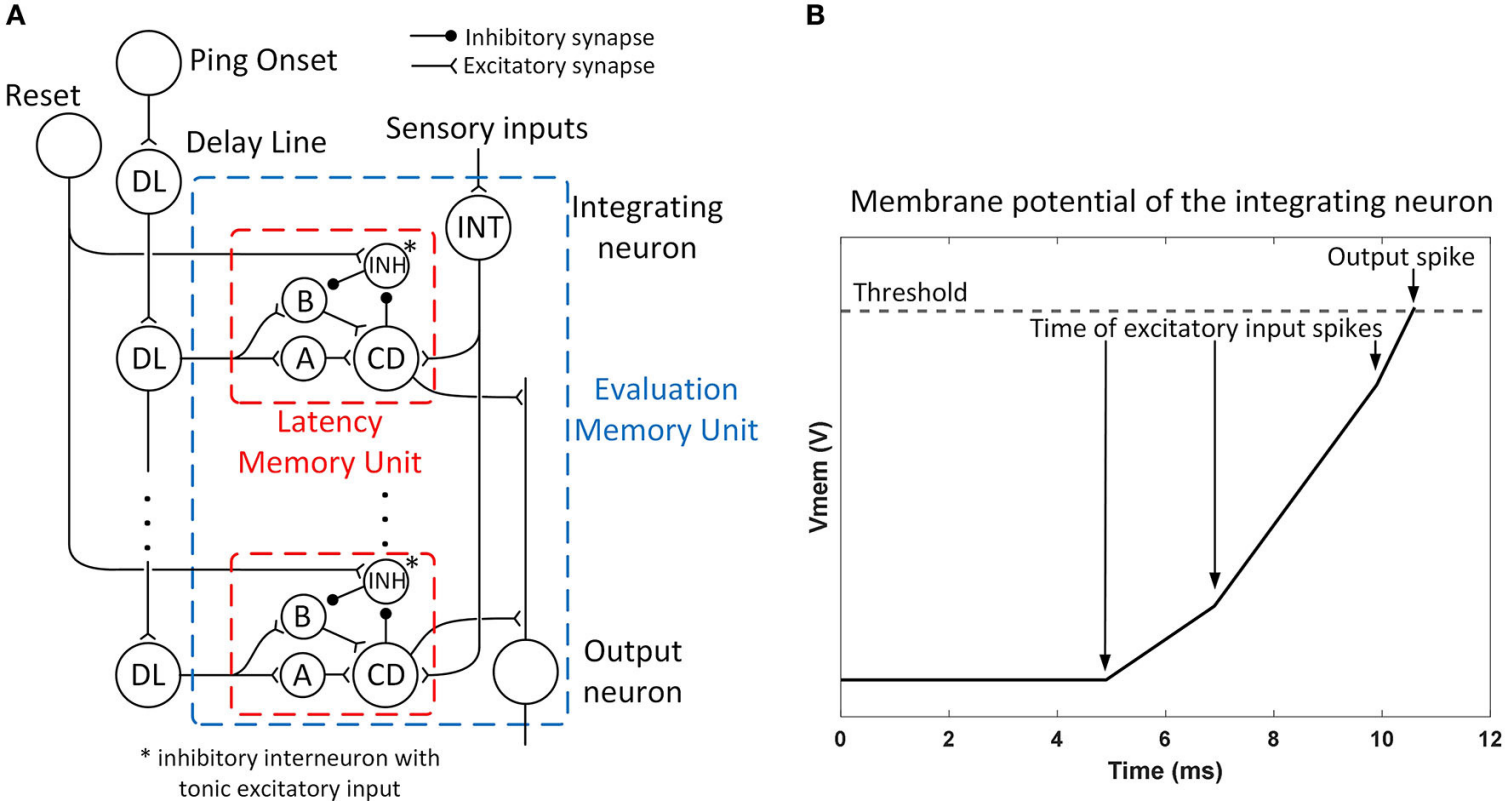
**(A)** The structure of an evaluation memory unit. The integrating neuron combines the spike train from sensory neurons into a single spike with a latency representing the risk of the corresponding path. The spike latency is then stored in an array of latency memory units. The memory can be reset by exciting the reset neuron. The output neuron combines the spikes from latency memory units into a single spike train and sends it to the action selection layer. **(B)** The mechanism of the integrating neuron. Each input spike triggers a step-excitation current that charges up the membrane potential *V*_*mem*_ and the currents from different sensory neurons accumulate. A spike is fired when *V*_*mem*_ crosses a threshold. An input spike that arrives earlier will result in a shorter spike latency. Input spikes after the first one also reduces the spike latency by increasing the charge speed.

The role of the evaluation memory (as described in Section Evaluation Memory) is to hold the spatially integrated value of immediacy along each path even when the sonar is interrogating a different direction and does not receive new sonar information for a given path. Given the spike latency representation, the output of the evaluation memory unit is a spike with a latency (following the sonar ping) that matches the previously observed latency (when the sonar was receiving new data). Each evaluation memory unit receives spikes with different latencies from the sensory neurons along a path and integrates them into a spike latency with the integrating neuron. An array of memory units along with a delay line detects and stores the occurrence of a spike at a particular latency and is then able to regenerate the spike upon later activation. The neural circuit of each evaluation memory unit is shown in Figure 7A.

An example of the mechanism of the integrating neuron is shown in Figure 7B. The integrating neurons are integrate-and-fire neurons and their membrane potentials simultaneously reset when the bat pings (*t* = 0). Whenever the integrating neuron receives a spike, a step-excitation current is turned on (for 20 ms), causing its membrane potential to rise. If the membrane potential reaches a threshold, the integrating neuron fires a spike. The spike latency is shorter when excitatory spikes arrive earlier (representing stronger inputs). The excitatory currents from different spikes are summated and can further reduce the latency of the spike.

For an integrate-and-fire neuron with a membrane capacitance *C*_mem0_, a spike threshold *V*_th0_, and *n* (*where n*≥1) step-excitation currents each with an amplitude of *E*_0_ activated at time *t*_1_, *t*_2_, …, *t*_*n*_, the latency of the spike *T*_eval_ is given by


(9) $$\begin{array}{c} T_{\text{eval}}=\frac{C_{\text{mem}0}V_{\text{th}0}+E_{0}\cdot \sum_{i=1}^{n}t_{i}}{n\cdot E_{0}} \end{array}$$

assuming that *t*_1_ ≤ *t*_2_ ≤ ⋯ ≤ *t*_*n*_ < *T*_eval_. Each input spike that arrives before the output spike reduces the spike latency, but the latency cannot be reduced below the latency of the first input spike. The spike latency *T*_eval_ represents the integration of immediacy of avoidance with a saturation limit similar to the second term in Equation 4, although the integration is different from simple addition. If the integrating neuron receives one or more spikes from the sensory layer, it will fire a spike with a latency of *T*_eval_ which resembles the evaluated risk of a path. Different from the risk calculation in the analog model (Equation 4), here a smaller latency means a larger risk value. The latency of the spike then needs to be stored in the evaluation memory.

An array of latency memory units and a delay line are used to store the latency of the spike from the integrating neuron and later generate a spike with the same latency when needed without the original sensory inputs. As is shown in Figure 7A, each evaluation memory unit has an array of latency memory units while the delay line is shared among all the evaluation memory units. The delay line is triggered by the onset of the sonar ping, and it generates spikes with different latencies which are sent to different latency memory units in different evaluation memory units. In this neural model, the delay line is implemented as neurons connected in series with excitatory synapses and each spike from the previous neuron causes the next neuron to fire with a fixed delay. The delays between neurons in the delay line affect the resolution of the latency memory.

As shown in Figure 7A, a latency memory unit consists of four neurons: two excitatory interneurons (A and B), a coincidence detector (CD), and an inhibitory interneuron (INH) with tonic excitatory input. Neurons A and B both take excitatory input from a neuron in the delay line, but neuron B also takes inhibitory input from neuron INH. Besides the tonic excitatory input, neuron INH is strongly inhibited by the CD neuron and strongly excited by a reset neuron. Without the input from the CD and the reset neuron, neuron INH fires tonically and keeps neuron B inhibited. The CD takes excitatory inputs from neuron A, neuron B, and the integrating neuron. For a CD to fire a spike, two spikes need to arrive at approximately the same time, meaning that two out of the three neurons exciting the CD need to fire simultaneously.

During the idle state before a sonar ping, neuron B is inhibited by neuron INH. When the bat pings, the ping onset starts the spike propagation in the delay line, and a spike with a certain latency will be sent to neurons A and B. Neuron A will be excited by the input spike and send a spike to the CD, whereas neuron B will not fire because it is strongly inhibited by neuron INH. At this point, only a spike from the integrating neuron with the same latency as the spike from the delay line will be able to trigger a spike from the CD. If this is the case, the CD will send a spike to neuron INH and keep it from firing again for the duration of the inhibitory synaptic input (around 300 ms). If the bat pings again while neuron INH is still inhibited, neurons A and B will both fire and the CD will fire again even without the spike from the integrating neuron. Since the CD fires again, neuron INH is kept inhibited for another interval, allowing the next sonar ping to cause neuron B to fire. Unless neuron INH is reset by the reset neuron or the bat doesn't ping for a long time, the memory of the spike latency from the integrating neuron is maintained and a spike with the same latency is reproduced after every sonar ping without any further sensory inputs.

The CD also sends an excitatory spike to the output neuron when it fires. The output neuron in each evaluation memory unit fires a spike with very little delay whenever it receives spikes from any of the latency memory units. Like an OR function, it combines all of the spikes from the delay-tuned neurons into a spike train. Due to the mechanism of the action selection layer, however, as will be described later in Section Motor Layer, only the latency of the first spike in the output spike train affects the calculation of the desirability. In the scenario where an evaluation memory unit with a stored spike latency receives new sensory input, only the spike with the shorter latency is meaningful to the next layer. Since a shorter spike latency represents a higher risk value, this behavior is consistent with the description earlier in Section Evaluation Memory that the final risk value is the maximum value between the decayed old risk and the new risk computed from the objects currently being sensed.

### Action Selection Layer

**Figure 8:**
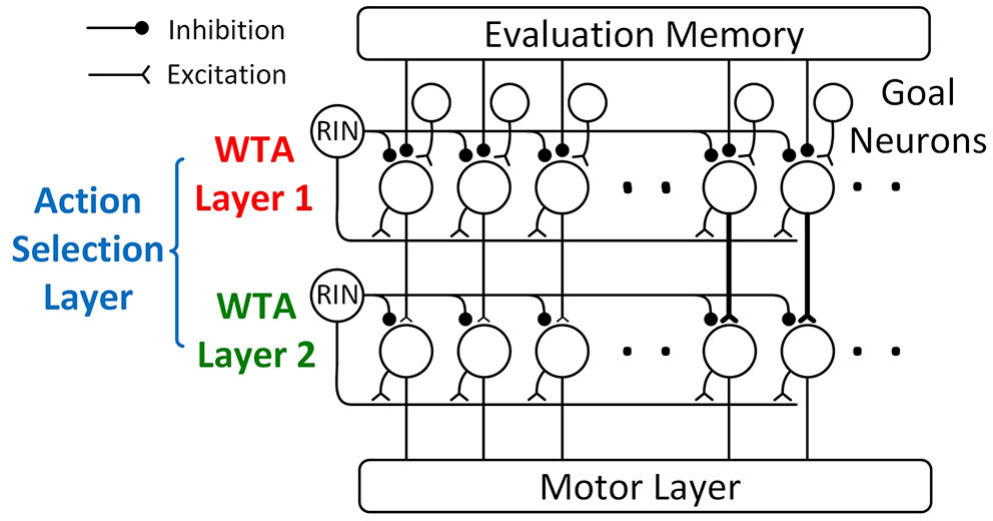
The action selection layer is composed of two WTA layers: WTA layer 1 and WTA layer 2. Both layers use a “race-to-first-spike” WTA mechanism, where the first neuron to spike excites a recurrent inhibitory neuron (RIN) to fire that suppresses other neurons within the layer from firing. Because WTA layer 1 can produce multiple winners that represent significantly different turning, WTA layer 2 is added to only allow the winners from the same ping direction group to go to the motor layer. In WTA layer 2, neurons toward the center (straighter paths) have a stronger synaptic connection from WTA layer 1 (represented by thicker synapses in the figure), giving those paths priority over paths toward the side (sharper turns).

The action selection layer consists of two WTA layers (Figure 8) with a similar structure, and both of the layers use a “race-to-first-spike” WTA mechanism to select the winning motor actions.

WTA layer 1 is similar to the temporal WTA circuit proposed in Horiuchi (2009). It compares the desirability of different paths using the latency of the spikes from the evaluation memory layer and selects the most desirable path with a “race-to-first-spike” WTA mechanism. WTA layer 1 consists of action selection neurons with an integrate-and-fire mechanism, a recurrent inhibitory interneuron (RIN), and a group of goal neurons (Figure 8). The numbers of action selection neurons and goal neurons are the same as the number of motor actions. The action selection neurons in WTA layer 1 receive weak excitatory input from the goal neurons that indicate the location of the goal. Each goal neuron connects to the field of action selection neurons with a Gaussian-shaped pattern of synaptic strengths (not all the connections are shown in Figure 8 for clarity). Only one of the goal inputs will fire a spike to indicate the path that leads to the goal. This excitatory connection corresponds to the Gaussian-shaped goal input in Equation 5 described in section action selection layer. The action selection neurons also receive weak excitatory input from the corresponding ping direction neuron through a delay neuron (“Delay” in Figure 6). Upon receiving a spike from the ping direction neuron, the delay neuron fires a spike after some delay to the action selection neurons associated with the same ping direction. This excitatory connection corresponds to the ping direction bias term in Equation 6. In addition to the connections from goal and ping direction neurons, all the action selection neurons receive passive excitatory currents that reflect the baseline desirability of different paths, corresponding to the “Constant Bias” term in Equation 6. This excitatory current could be either from a neuron firing tonically or intrinsic membrane currents (Häusser et al., 2004).

**Figure 9:**
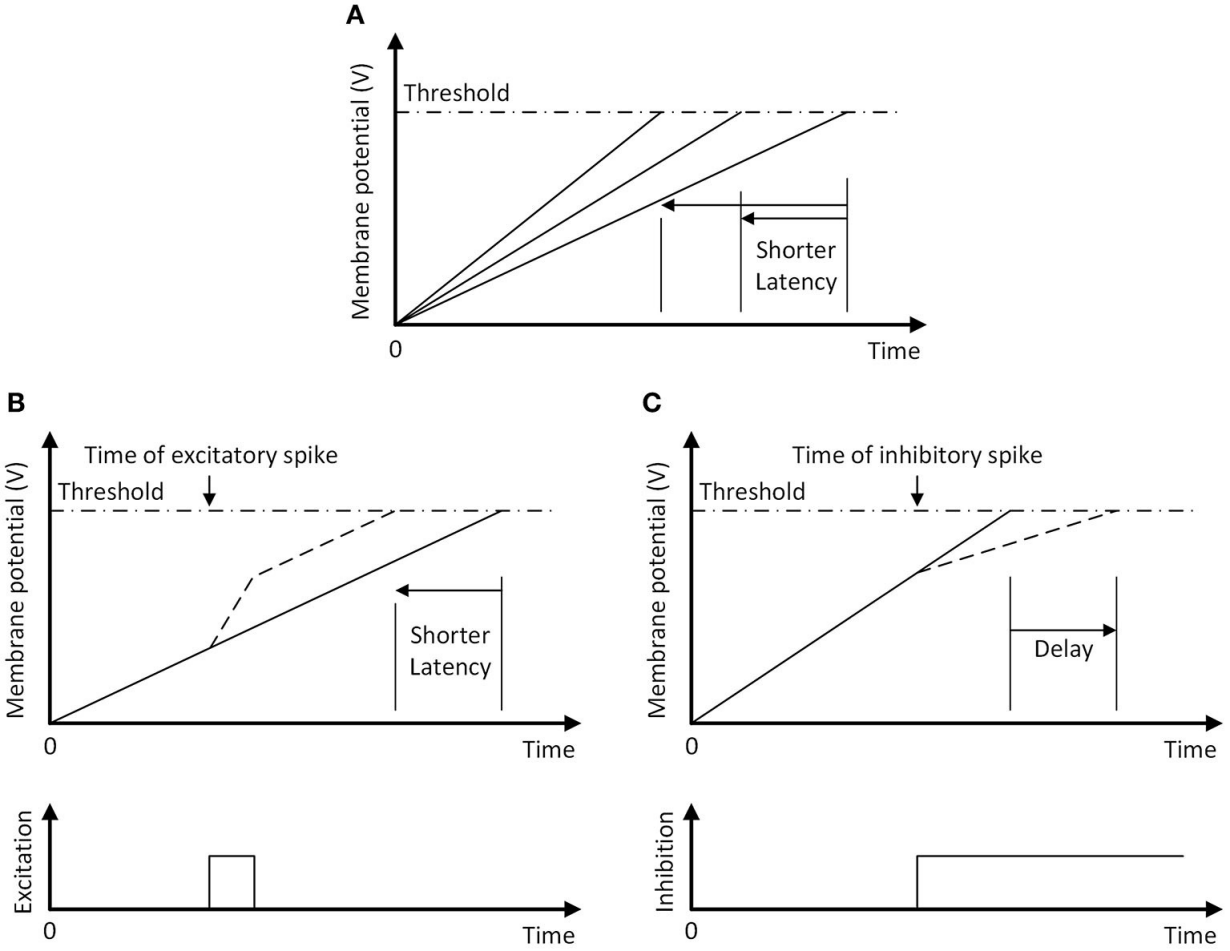
The temporal WTA mechanism with an integrate-and-fire neuron. The neuron fires a spike when its membrane potential reaches the threshold (dash-dotted line). **(A)** Following a reset, increasing the passive excitatory currents increases the charging rate of the membrane potential and shortens the latency of the spike. **(B)** A pulse of excitatory current increases the membrane potential, shortening the spike latency (dashed line). **(C)** A long-lasting step-inhibition current increases the latency of the spike (dashed line) or prevents firing altogether. An inhibitory spike that arrives earlier produces a longer delay in firing.

When a sonar ping is emitted, the ping onset neuron simultaneously resets (i.e., strongly inhibit and then release) all of the action selection neurons. The passive excitatory currents can then be inversely expressed in the spike latency across the field of neurons (Figure 9A). In the absence of other inputs, the neuron that receives the strongest excitatory current will integrate to the threshold first and is the winner, meaning that the motor action with the largest constant bias will win. The excitatory spikes from the goal and ping direction neurons increase the membrane potential, thus decreasing the amount of charge that the IF neurons need to reach the firing threshold and making them more likely to win (Figure 9B).

Each evaluation memory unit is connected to all of the action selection neurons through inhibitory synapses that activate a long-lasting step-inhibition current if a spike arrives (not all connections are shown in Figure 8 for clarity). The synapses have a Gaussian distribution of synaptic strengths with the peak centered on the synapse connecting the neurons representing the same path. Different synaptic weights mean different amplitudes of the activated inhibitory current. This way of connecting the evaluation memory and the action selection layer corresponds to the Gaussian inhibition term in Equation 5. The standard deviation of the Gaussian distribution, however, is small (σ_*c*_ = 1 in the simulation) and in practice synapses 3σ_*c*_ away from the center can be pruned without affecting the performance. The inhibitory current from a synapse saturates when the synapse receives a spike and any following spikes to the same synapse will not increase the amplitude of the inhibitory current. The saturating current from a synapse is the reason why only the latency of the first spike from an evaluation memory unit affects the computation of desirability.

The accumulated inhibitory currents from different synapses slow the rate of charging of the action selection neuron and the time to spike will increase as inhibitory inputs start earlier (Figure 9C). Because the spike latency from the evaluation memory is inversely related to the risk of different paths, a path with a higher risk will result in an earlier activation of inhibitory currents and thus increase the spike latency of the action selection neurons. In combination with passive currents and excitatory spikes from the goal and ping direction neurons, the action selection neuron that fires the first spike indicates the most desirable path.

For an action selection neuron with a membrane capacitance *C*_mem_, a spike threshold *V*_th_, a passive excitatory current *I*_exc_, a sum of injected charge from goal neurons *Q*_goal_ and ping direction neurons *Q*_dir_, and *n* step-inhibitory currents activated at time *t*_1_, *t*_2_, …, *t*_n_, the latency of the spike *T*_WTA_ is given by


(10) $$\begin{array}{c} T_{\text{WTA}}=\frac{C_{\text{mem}}V_{\text{th}}-Q_{\text{goal}}-Q_{\text{dir}}-\sum_{i=1}^{n}I_{i}\cdot t_{i}}{I_{\text{exc}}-\overset{i=1}{\underset{n}{\sum^{\text{​}}}}I_{i}} \end{array}$$

where *I*_*i*_ is the amplitude of the inhibitory current activated at time *t*_*i*_. It is assumed that *t*_1_ ≤ *t*_2_ ≤ ⋯ ≤ *t*_*n*_ < *T*_WTA_, meaning the spikes that arrive after the neuron fires are ignored. It is also assumed that $I_{exc}-\sum_{i=1}^{n}I_{i}>0$, or else no spike is generated. As is shown in Equation 10, a larger passive excitatory current decreases the spike latency by increasing the denominator while stronger excitatory inputs from goal and ping direction neurons decrease the latency by reducing the numerator. Earlier arrival of inhibitory spikes (smaller *t*_*i*_) increases the latency by increasing the numerator. Although the amplitudes of the inhibitory currents *I*_*i*_ affect both the denominator and the numerator, stronger inhibitions that arrive at the same time still increase the spike latency. In a cluttered environment where obstacles are blocking many paths, the sum of the inhibitory currents could exceed the sum of excitatory currents and prevent the neuron from firing a spike at all.

The first spike from the action selection neurons then causes a recurrent inhibitory neuron (RIN) to fire which recurrently inhibits every action selection neuron and prevents them from firing. Due to the non-zero latency of the RIN and the slow activation of inhibitory synapses, there is a delay between the first spike from the action selection neuron and the start of the recurrent inhibition. Because the passive excitatory current is weak and the excitatory inputs are short, the time it takes for the action selection neuron to fire a spike is much longer than the delay of the recurrent inhibition. As a result, each of the action selection neurons either fires a single spike or produces no spike at all. However, any spikes that occur before the inhibition is effective will pass through the WTA mechanism and multiple winners can appear at the output. This behavior is similar to the behavior of a *k*-WTA network which selects the *k* largest values. Although *k*-WTA networks have been shown to be useful in robot systems (Peng et al., 2021; Qi et al., 2021), having multiple winners at the output of the WTA layer in our proposed neural network could cause problems in both the head motor layer and the body motor layer. In our model of the motor layers, we assume that multiple activations of the motor neurons would cause averaging of motor actions. While multiple winners that encode nearby head directions and motor actions do not necessarily cause problems, winners representing significantly different paths will. To solve the problem with multiple winners, a second WTA layer (“WTA Layer 2” in Figure 8) is added to ensure that the winners sent to the motor layer are within the same ping direction group by eliminating some of the winners.

The structure of WTA layer 2 is the same as WTA layer 1 except for the input synapses. The action selection neurons in WTA layer 2 take one-to-one excitatory connections from the neurons in WTA layer 1. All of these connections are strong enough to fire the excitatory neurons with a single spike, and stronger synaptic connections produce spikes with shorter delays. The synaptic strengths are the same for neurons within each of the 5 ping direction groups but differ from group to group to create 5 different delays. The values of the delays are designed so that the minimal difference between delays is longer than the delay of the recurrent inhibition. As a result, although WTA layer 2 can still produce multiple winners, it is guaranteed that the winners are from the same ping direction group. The values of the synaptic strengths decide the priority of each ping direction group. It is beneficial in terms of safety and energy cost to favor slow turns over sharp turns, so in this model, the synaptic strengths in the middle group are set to be the highest, followed by the middle-left group, the middle-right group, the left group and then the right group.

### Motor Layer

The function of the motor layer is to produce the motor actions needed to orient the head of the bat (head motor layer) or to turn the body (body motor layer) to follow a selected path.

Five head motor neurons representing five head (i.e., ping) directions receive excitatory input from the neurons in the action selection layer that are associated with the same ping direction (Figure 6). Upon receiving a spike, a head motor neuron becomes active and orients the head of the bat in the associated ping direction for the next sonar ping, consistent with the proposed attentional system described in Section Motor Control. Because only the neurons in the same ping direction group can win in the action selection layer, as described in Section Action Selection Layer, no more than one head motor neuron will be active at the same time.

Each body motor neuron is associated with a path, and when it fires, it produces the correct body/wing changes to execute the turn rate needed for its associated path. When multiple body neurons are active, the bat is modeled to produce the averaged turn rate and execute the averaged path, similar to the population coding hypothesis seen in other animals (Lee et al., 1988). The body motor neuron receives excitatory input from the action selection neuron associated with the same path and inhibitory input from an interneuron inhibited by the recency memory. Each recency memory is associated with a ping direction, and it is a decaying memory that stores the recency of its associated ping direction. The recency memory becomes active when a ping direction neuron fires, and it inhibits the interneuron for some duration until decayed back to its inactive state. The duration of the memory is decided by the duration of the excitation from the ping direction neurons. In this scenario, the inhibited interneuron can no longer inhibit body motor neurons and they are allowed to fire upon receiving spike input from the action selection layer. Through this disinhibition mechanism, a bat can turn its body to follow a winning path only when it has recent information in its associated ping direction.

In the situation where none of the action selection neurons fire a spike in a certain time window after the outgoing ping, all paths are undesirable for the bat to follow and the emergency “turnaround” maneuver will be executed as described in Section Motor Control. It is achieved by adding an “emergency” neuron in the action selection layers with a fixed spike latency and connecting it to a motor neuron responsible for the emergency maneuver. The emergency neuron will be inhibited if any other action selection neurons fire a spike before it does, creating a time window where a path is good enough to follow.

## Experiment Results

### Simulation of the Analog Model

**Figure 10:**
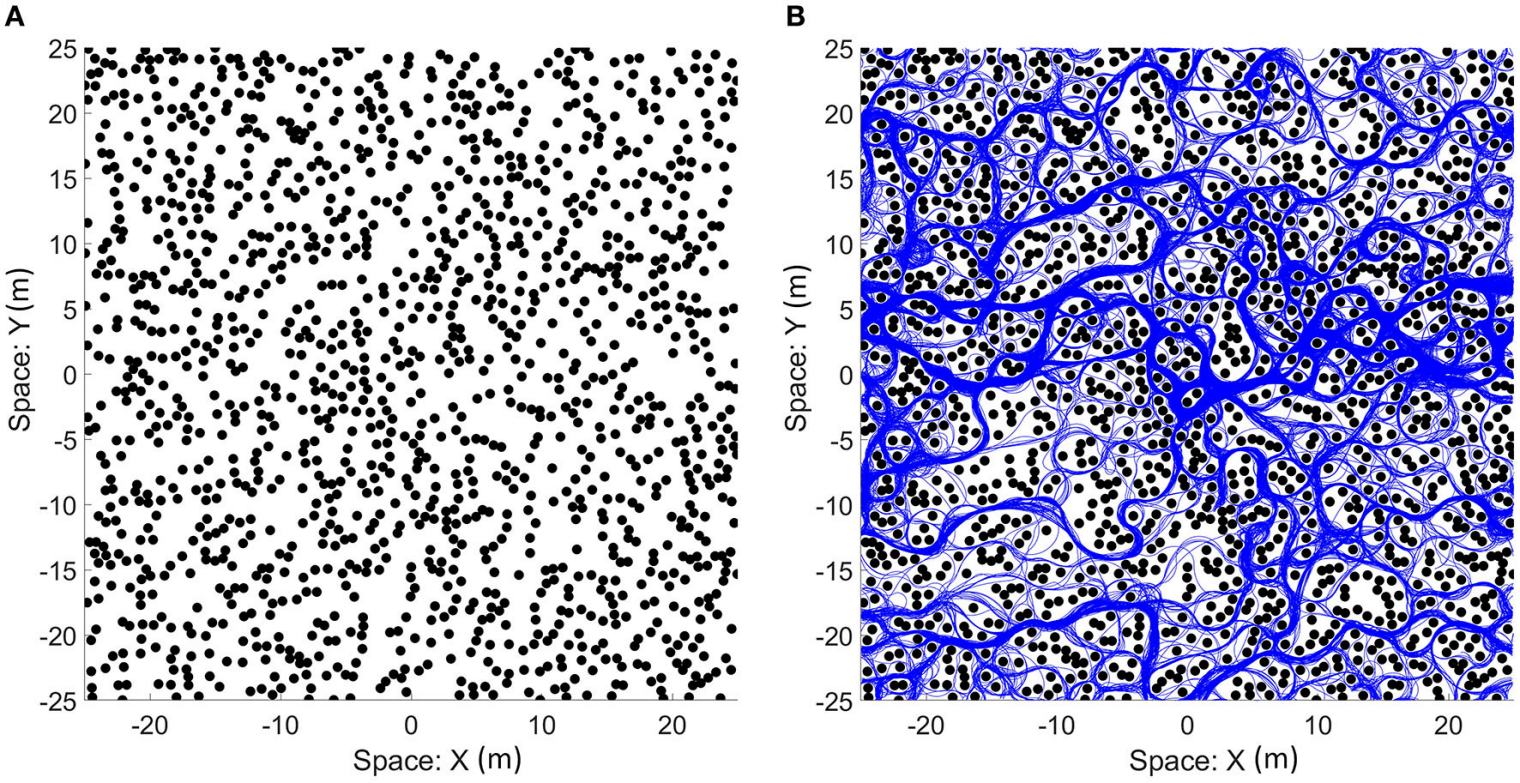
**(A)** An example of the field with randomized locations of obstacles. One thousand four hundred obstacles (black circles) were placed in a 50 × 50 m area. The size of the obstacles shown in the figure is the size of the zone of collision (combination of the sizes of the bat and the obstacle). The space of the field can be mapped onto a torus to simulate a bat traveling on an infinite plane as the bat can sense and travel through boundaries. **(B)** The paths that the bat traveled (blue line) on the field. Although open areas were traveled more often by the bat, most of the viable paths were visited.

We focus our simulation on the bat traveling in a field with densely placed obstacles to test the capabilities and characteristics of the analog model. 1,400 identical obstacles are placed randomly in a 50 × 50 m field with two-dimensional periodic boundary conditions (Figure 10A). The objective of the bat is to travel in the leftward direction without colliding with any obstacles. We provided a dynamic goal input to drive the bat to a goal destination at the left boundary (X = 0) with the same vertical position (same Y coordinate) and updated it at each time step according to the position of the bat. When the bat crosses the left boundary, it will reappear at the right boundary with the same orientation and speed before the crossing. An example of the traveled path in a simulation is shown in Figure 10B and it shows that the bat visited most of the viable paths instead of repeatedly traveling on a few paths.

In the simulations, the bat used the accurate locations of the obstacles whose centers were in the current FOV. The occlusion of distant obstacles was not simulated. The FOV function (detectable distance vs. relative angle to the head direction) used in the simulation is a Gaussian distribution with σ = 30 with a maximum distance *R*_MAX_ = 5 *m*, resulting in a half-maximum width of 70.7. For big brown bats, the half-power beamwidth (the angular width of the beam pattern at the 3 dB cutoff points) of their emitted ultrasonic signal at 35–40 kHz is 56 to 80 (Ghose and Moss, 2003; Gaudette et al., 2014). Although the FOV of a typical sonar system is not equivalent to the beampattern of the emitted sound, we approximated them to be similar and designed the FOV with a similar shape. The bat emitted sonar pings at a maximum rate of 5 Hz in the simulation with the exception in section 4.1.4 where the maximum sensing rate is increased to test its influence on the performance. Big brown bats can send out sonar pulses to sense obstacles at a rate of up to 90 Hz (Sändig et al., 2014) and turn their heads to ping in a different direction in <30 ms (Surlykke et al., 2009). We used a much lower maximum sensing rate in the simulation to push the limits of the model and test its capabilities. It is assumed that there are no errors in the steering action and the bat was able to follow the winning path exactly.

Defining performance metrics for collision avoidance is dubious because it must always be evaluated in the context of another movement-inducing behavior (e.g., goal-seeking) and most comparisons are done between performance measures such as path length and travel time. Furthermore, collision avoidance performance is strongly correlated with the limitations of the sensors. Comparisons to other algorithms are problematic if their algorithms do not also use narrow-FOV sensors. Nonetheless, we have defined a performance measure to understand the impact of features and parameters in the proposed model. We use the goal-seeking behavior and measure how long the bat can move toward the goal without a collision. Specifically, the number of unique obstacles that the bat detected, but did not collide with, is counted as the number of avoided obstacles. Only unique obstacles were counted because the bat could be trapped temporarily in a local area and the obstacles around the bat should not be counted more than once in this scenario. Whenever the bat crossed the left boundary, the bat was considered to have entered a new environment and all the obstacles could be counted again. The simulation ends if one of the three scenarios happened: (1) the bat collided with an obstacle; (2) the bat has not crossed the left boundary in a long time (2,500 s) suggesting that it is trapped; (3) the simulation has reached a time limit (250,000 s). In the second and third scenarios, the simulation restarts with a different random seed, and the number of avoided obstacles is accumulated. Whenever the bat collided with an obstacle, the accumulated number of avoided obstacles before the collision is recorded and is considered as a sample.

#### Density of Obstacles

**Figure 11:**
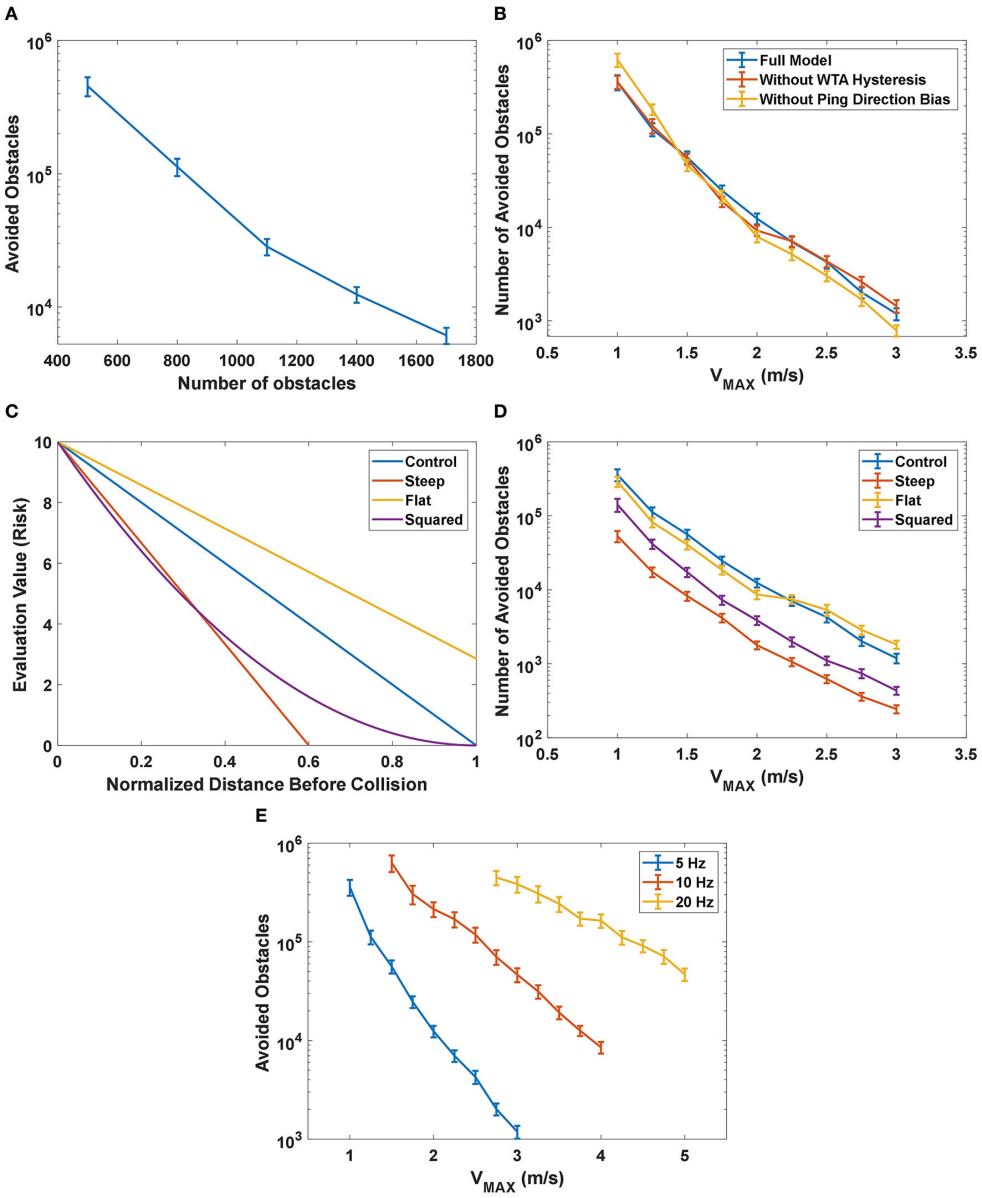
**(A)** The performance of the proposed model as a function of the number of obstacles in the field. The number of avoided obstacles increased, approaching infinity, as the number of obstacles decreased. **(B)** The simulation results of the effect of different terms in the desirability function under different maximum speed (*V*_MAX_) settings. The control configuration included all the terms described in Equation 6 while the configuration without WTA hysteresis had *H* = 0 and the group without ping direction bias had *P* = 0. The group without WTA hysteresis had similar performance at most of the speed settings compared to the control group, while the group without ping direction bias had significantly better performance at lower speeds (*V*_MAX_ ≤ 1.25 *m*/*s*) and significantly worse performance at higher speeds (*V*_MAX_≥2 *m*/*s*). **(C)** The shape of different immediacy functions tested in the experiment. **(D)** The performance of the model with different immediacy functions. The results from the “Steep” group and the “Squared” group showed that reducing the significance of distant obstacles negatively impacted the performance across all speed settings, and thus indicated that distant obstacles were important to the decision-making process of the model. The comparison between the control group and the “Flat” group showed that increasing the significance of distant obstacles could be beneficial at higher speed. **(E)** The effect on the performance from changing the maximum rate of sonar pings. The performance not only was significantly increased with increased rate of pings but also declined more slowly with the increase in the maximum speed.

To test how the proposed model performed with different densities of obstacles in the field, simulations were run with different numbers of obstacles ranging from 500 to 1,700. Because the simulated area was constant, changing the number of obstacles in the 50 × 50 m field is equivalent to changing the density of obstacles. The simulation results are shown in Figure 11A. As expected, the performance increased as the density decreased.

#### Desirability Function

To test the effect of different terms in the desirability function (Equation 6) under different maximum speed settings, we compared the performance between a control configuration of the proposed model across different speed settings (i.e., control group), a configuration without WTA hysteresis and a configuration without ping direction bias (Figure 11B). The control simulations used all the terms of the desirability function described in Equation 6, while the group without WTA hysteresis excluded the hysteresis term (*H* = 0) and the last group excluded the ping direction bias term (*P* = 0). The average number of avoided obstacles is calculated based on 200 samples under different maximum speed (*V*_MAX_ in Equation 1) settings and the 95% confidence intervals for each configuration are shown as error bars in Figure 11B.

The group without WTA hysteresis had significantly worse performance (*p* < 0.05) only at *V*_MAX_ = 2 *m*/*s* compared to the control group. Hysteresis is generally introduced in a WTA layer to prevent the winner from oscillating between a few similar candidates due to noise in the system. We believe that one of the main reasons why WTA hysteresis did not have a significant impact on the performance is because sensory or other noise is not included in the simulation.

A comparison of performance shows that the group without ping direction bias had significantly better performance (*p* < 0.05) than the control group when *V*_MAX_ ≤ 1.25 *m*/*s* and has significantly worse performance (*p* < 0.05) when *V*_MAX_≥2 *m*/*s*. As mentioned in section action selection layer, the function of the ping direction bias is to encourage the bat to select the path with the most recent information when several paths have similar risks. At low speeds, the evaluation memory of the paths from different ping directions is relatively accurate and adding the ping direction bias frequently prevents the bat from selecting the actual “best” path, thus dropping the performance. At high speeds, however, the evaluation memory may be wildly inaccurate and selecting a slightly better path according to the memory could be more dangerous than selecting a “good” path with recent and accurate information. The results suggest that features in the desirability function provide benefits in different situations and adding a speed-dependent controller on the weights of different features could help improve performance.

#### Immediacy Function

We tested and compared different functions representing the immediacy of avoiding an obstacle. As a reminder, the immediacy of avoiding a blocking obstacle *j* on a path *p* is represented as a function (*f*).


(11) $$f_{p}(j)=E_{\text{MAX}}\;\cdot \;\left[1-\frac{rp\prime (j)}{R_{\text{MAX}0}\cdot \gamma_{p}}\right]$$

where *E*_MAX_ is a constant representing the maximum value of *f*_*p*_ and is set to 10 for all the different configurations in this experiment. $r^{\prime }{}_{p}\left(j\right)$ is the distance that the bat can travel along path *p* before colliding with a blocking obstacle *j*. The distance is then normalized with the speed profile γ_*p*_ to represent the time before colliding with the obstacle.

The results from this experiment are shown in Figures 11C,D. The control group had the same function of Equation 11 with *R*_MAX0_ = *R*_MAX_ = 5 and it decreased linearly with the distance to the obstacle normalized by the speed profile γ_*p*_. Because *R*_MAX0_ = *R*_MAX_, the immediacy function decreased to 0 when the normalized distance $\frac{rp\prime (j)}{\gamma_{p}}$ is 1, as described earlier. The “Steep” group had *R*_*MAX*0_ = 3, thus creating a steeper linearly-decreasing function compared to the control group. The “Steep” function was rectified to 0 when it dropped below 0, which means that more distant obstacles were simply ignored. The “Flat” group had *R*_*MAX*0_ = 7, resulting in a flatter function that included the contributions of a blocking obstacle even when it was distant. As a tradeoff, the differences between obstacles at different distances were reduced. The “Squared” group had a function as $f_{p}(j)=E_{MAX}\;\cdot \;{\left[1-\frac{rp\prime (j)}{(R_{\text{MAX}}\cdot \gamma_{p}}\right]}^{2}$, which emphasizes close obstacles without completely ignoring distant obstacles.

As shown in Figure 11D, the number of avoided obstacles in the “Steep” group was significantly lower than all other groups across all speed settings, followed by the “Squared” group with the second-worst performance. It showed that throwing away or reducing the significance of the information about distant obstacles severely reduced performance, indicating that the proposed model could make good use of this information in its decision-making process. The “Flat” performed significantly worse (*p* < 0.05) when 1.5 *m*/*s* ≤ *V*_MAX_ ≤ 2 *m*/*s* but performed significantly better (*p* < 0.05) at speeds higher than 2 *m*/*s*. Distant obstacles present a greater threat at higher speeds, thus the steering decisions could benefit from emphasizing them. The results suggest that adjusting the evaluation function accordingly at different speed settings could improve performance.

#### Maximum Ping Rate

In the previous experiments, the maximum ping rate was set to be 5 Hz. As mentioned before, echolocating bats can emit outgoing sonar pings up to a rate of 90 Hz (Sändig et al., 2014). In this experiment, we increased the maximum rate of sonar pings to 10 and 20 Hz to test its effect on the performance.

As shown in Figure 11E, the performance improved significantly with increased ping rates. Increasing the ping rate also helped their performance decline more slowly with the increase in speed settings, indicated by a lower slope of 10 and 20 Hz curves compared to that of the 5 Hz curve. A higher rate of pings allowed the bat to travel at a much higher speed while maintaining its performance.

### Spiking Neural Model Simulation

The proposed spiking neural model was implemented in Python using a combination of Hodgkin–Huxley neurons (Hodgkin and Huxley, 1952) and leaky integrate-and-fire (LIF) neurons. The Hodgkin-Huxley model was applied to as many neurons as possible to show the biological plausibility of the proposed spiking neural network, and simpler LIF neuron models were used to implement sensory neurons in the sensory layer, integrating neurons in the evaluation memory and WTA neurons in the action selection layer. The sensory neurons were implemented in LIF neurons to speed up the simulation because of their large number and their simple function (fire a spike upon receiving a spike). Implementing them with the LIF neurons does not change their function significantly compared to the implementation with Hodgkin-Huxley neurons. The integrating neurons and the WTA neurons used LIF models because their function includes integrating input spikes over a longer time interval (~40 ms). In total, there are 2,931 LIF neurons and 2,078 Hodgkin-Huxley neurons in the neural model.

The synaptic connections to the Hodgkin-Huxley neurons were implemented with a time-dependent conductivity function *g*_syn_(*t*). The synaptic current is calculated as


(12) $$\begin{array}{c} I_{\text{syn}}(t)=g_{\text{syn}}(t)\cdot [v(t)-E_{\text{syn}}] \end{array}$$

The reversal potential *E*_syn_ and the function *g*_syn_(*t*) can be used to describe different types of synapses. For excitatory synapses, *E*_syn_ is set to 10 mV whereas for inhibitory synapses, *E*_syn_ is set to −70 mV. In this simulation, an alpha function was used to describe the synaptic conductance *g*_syn_(*t*) as:


(13) $$\begin{array}{c} g_{\text{syn}}(t)={\bar{g}}_{\text{syn}}\cdot (t-t_{0})(\frac{e}{\tau })e^{-\frac{\left(t-t_{0}\right)}{\tau }}\;\cdot \;\Theta (t-t_{0}) \end{array}$$

where τ is the time constant, ${\bar{g}}_{\text{syn}}$ is the maximum conductance, *t*_0_ is the arrival time of a pre-synaptic spike and Θ(*t*) is the Heaviside step function. The synaptic connections to LIF neurons are simplified as current injections, *I*_syn_LIF_(*t*), which are described by a boxcar function as


(14) $$\begin{array}{c} I_{\text{syn}\_\text{LIF}}(t)={\bar{I}}_{\text{syn}}\cdot [\Theta (t-t_{0})-\Theta (t-t_{0}-T_{\text{syn}})] \end{array}$$

where ${\bar{I}}_{\text{syn}}$ is the amplitude of the synaptic current and *T*_syn_ is the duration of the synaptic input.

Each sensory map in the sensory layer consists of 471 neurons representing locations of up to 25 ranges at 0.2-meter increments (from 0.2 to 5 m) and 31 angles at 4-degree increments (from −60° to 60°). Big brown bats (*Eptesicus fuscus*) are shown to be able to discriminate range differences as small as 1 cm (Simmons, 1973) and angular differences of 6° (Peff and Simmons, 1972), while other species such as the greater spear-nosed bat (*Phyllostomus hastatus*) can differentiate objects that are 4° apart. We used a relatively small number of neurons to represent the range because a higher range resolution significantly increased the total number of neurons and slowed the simulation while not significantly improving the performance of obstacle avoidance. The neurons that would represent locations outside of the field-of-view of the sonar were removed, thus the number of neurons in each map is smaller than the total number of possible combinations of ranges and angles. During the simulation, if an obstacle is within the sonar field-of-view when the bat pings, the obstacle will cause the sensory neuron with the closest receptive field in the head map to fire a spike.

**Figure 12:**
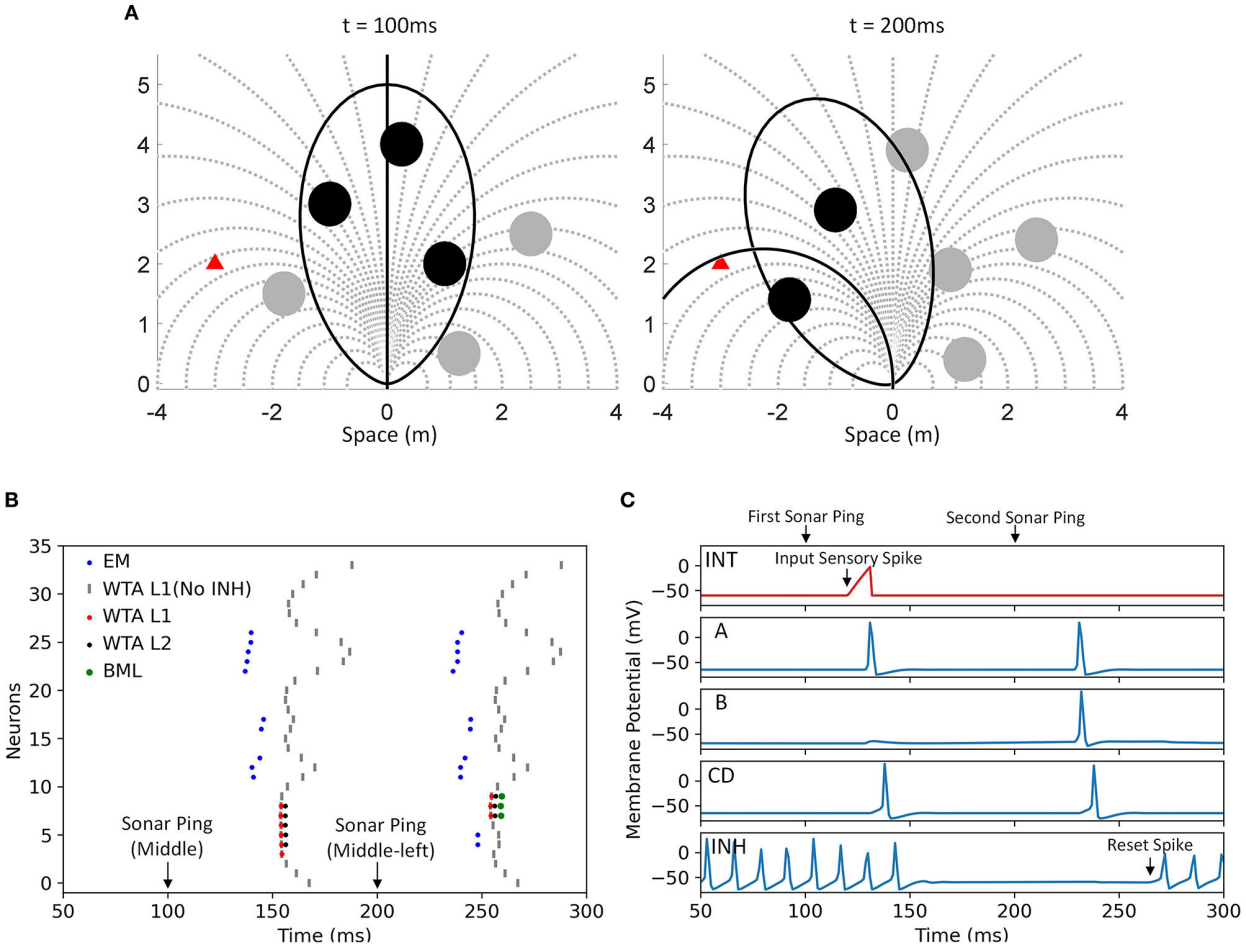
**(A)** The bat is in an environment with 6 obstacles (filled circle) and a desired destination (filled triangle in red) without prior information of the environment. The environment is the same as the example given in Figure 5 for a clearer comparison between the analog model and the spiking neural model. It is assumed that it was flying along a straight path (solid straight line) and it directed its first sonar ping to the front (ellipsoidal FOV shown in solid line). The circles of the obstacles are drawn with the size of the zone of collision to show the paths that they are blocking. The bat has a limited selection of motor choices (dotted line, 33 in total) and each of the motor actions corresponds to a neuron in the spike raster plot below. The bat sent the first sonar ping at *t* = 100 ms and a second one at *t* = 200 ms. The detected obstacles (filled circle in black) from each of the sonar ping caused the corresponding sensory neurons to fire. **(B)** The spikes from the output neurons in the evaluation memory (EM), the WTA neurons in the action selection layer and the neurons in the body motor layer (BML) were shown. The spike latency of each EM neuron represents the “risk” value of a path, and the spike latency of each WTA neuron represents the desirability of a path. The neurons with smaller indices represent paths to the left and the neurons with larger indices represent paths to the right. The spikes from an imaginary WTA layer 1 without recurrent inhibition are also plotted (gray vertical line) for a better demonstration of the impact that the spikes from the EM had on the action selection layer. **(C)** The membrane potentials of the neurons in the 9th latency memory unit (LMU) and the integrating neuron (INT) for path 24 are shown to demonstrate the working mechanism of the evaluation memory. The integrating neuron was implemented as a LIF neuron (red curve) and the neurons in the LMU (neurons A, B, CD and INH) were implemented using Hodgkin-Huxley model (blue curve).

The simulation of the proposed spiking neural network was implemented in Python and run on a CPU (Intel Xeon Cascade Lake) with a time step of 0.02 ms. A small time step was needed to ensure the correct simulation of the Hodgkin-Huxley neurons. The synaptic weights were fixed and calculated before the start of the simulation. An example of the simulation is shown in Figure 12. After the first sonar ping, the EM combined information from the sensory layer and produced three groups of spikes (blue dots in Figure 12B) whose latencies represent the risk values of the paths that were blocked by the three detected obstacles. Smaller latencies from the EM represent larger risk values. The EM spikes with smaller latencies caused larger delays in the firing of the WTA neurons (gray vertical lines), making those paths less desirable and less likely to win in the temporal WTA. Because several neurons in WTA layer 1 fired simultaneously, there were multiple winners (red dots). The biased WTA (layer 2) selected the winners that were closer to the center and in the same ping direction group (black dots).

The spikes from the winners, which belong to the middle-left group, caused the middle-left head motor neuron to fire a spike (not shown). As a result, the bat turned its head to the middle-left direction for the next sonar ping (right panel in Figure 12A). Because the bat did not have recent sensory information in the middle-left direction, no body motor neurons in the BML fired and the bat kept its original trajectory. The bat also maintained the memory of the spike latencies from the detected obstacles in the EM.

After the second sonar ping at 200 ms, the output neurons in the EM fired spikes in response to the two newly detected obstacles (EM neurons 4–5 and 11–13). At the same time, the neurons reproduced the spike latencies from the previously detected obstacles that were no longer in the field of view (EM neurons 16–17 and 22–26). Because the three winners from the action selection layer were all from the middle-left direction and the bat had recent information in this direction, the BML neurons fired spikes (green dots) and the bat executed the path averaged from the fired BML neurons (right panel in Figure 12A).

The membrane potentials of the neurons in an activated latency memory unit in this example are shown in Figure 12C to demonstrate the mechanism of the evaluation memory. The A neuron was excited by a delay line neuron (not shown) and fired a spike with a fixed latency after every sonar ping. Although the B neuron was excited by the same delay line neuron, it did not fire after the first sonar ping because it was inhibited by the tonically firing inhibitory interneuron (INH). Because a blocking obstacle was detected after the first sonar ping, the integrating neuron (INT) received a spike from the sensory layer and produced a spike with a latency representing the immediacy of avoiding that obstacle. When the spike from the INT neuron and the spike from the A neuron arrived at the coincidence detector (CD) neuron within 3 ms of each other, the CD neuron fired a spike which excited the output neuron of the evaluation memory unit. The spike from the CD neuron also inhibited the INH neuron and suppressed its firing for a long duration (~150 ms), releasing neuron B from its inhibition.

After the second sonar ping, the B neuron fired a spike at the same time as the A neuron because they were excited by the same delay line neuron. The coincidence of the spikes from A and B neurons caused the CD neuron to fire again and thus the same spike latency was reproduced. The spike from the CD neuron suppressed the INH neuron again and “extended” the latency memory. In this example, because the bat made a motor decision after the second sonar ping, all the latency memories were reset by an excitatory spike to the INH neuron (“reset spike” in the bottom panel in Figure 12C) that was strong enough to overcome the previous inhibition from the CD neuron and caused the INH neuron to start firing again. Once the INH neuron is firing, the B neuron is suppressed, and the CD neuron will no longer fire without the input from the INT neuron. Hence, the latency memory is “erased.”

**Figure 13:**
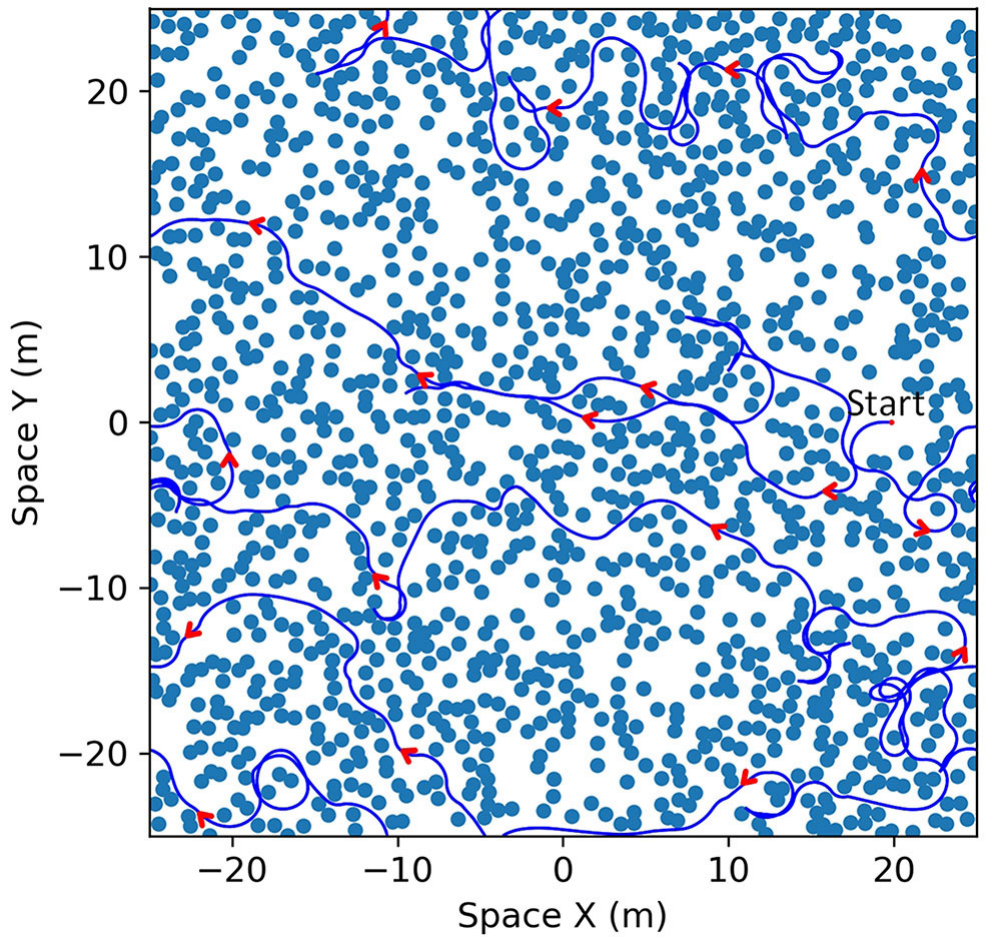
An example of the bat traveling at a maximum speed of 2 m/s in a dense forest using the proposed spiking neural model with a spike-timing representation. One thousand four hundred obstacles (filled circles in blue) were placed in a 50 × 50 m area. The size of the obstacles shown in the figure is the size of the zone of collision (combination of the sizes of the bat and the obstacle). The parameters of the bat were kept the same as the parameters used in the simulation of the analog model in section simulation of the analog model, except for the maximum ping rate which was set to 10 Hz in this simulation. The field has two-dimensional periodic boundary conditions to allow the bat to travel as if in an infinitely large space. A goal input is provided to drive the bat to a goal destination at the left boundary (X = 0) with the same vertical position (same Y coordinate). The paths that the bat traveled on the field are shown as blue curves.

The neural network was also simulated in the same environments used in the simulation of the analog model described in Section Simulation of the Analog Model. One of the simulation results is shown in Figure 13 where the bat successfully reached the left boundary 4 times without colliding with any obstacles.

Due to the complexity of the Hodgkin-Huxley model, simulating the proposed spiking neural network on a CPU is extremely slow (~10^5^ times slower than simulating the analog version), and collecting enough data to do performance analysis as in Section Simulation of the Analog Model is impractical with the current implementation. The short simulation run shown in Figure 13 took more than 5 days to complete. A neuromorphic VLSI implementation, however, could vastly improve the running speed of the proposed spiking neural network and can allow it to run in real-time on a mobile platform. It is important to note that it is not necessary to use the Hodgkin-Huxley model in the neural network and it is only used to show the biological plausibility of the network with more realistic neuron characteristics. The proposed network can produce similar results using simpler neural models.

### Robot Implementation

**Figure 14:**
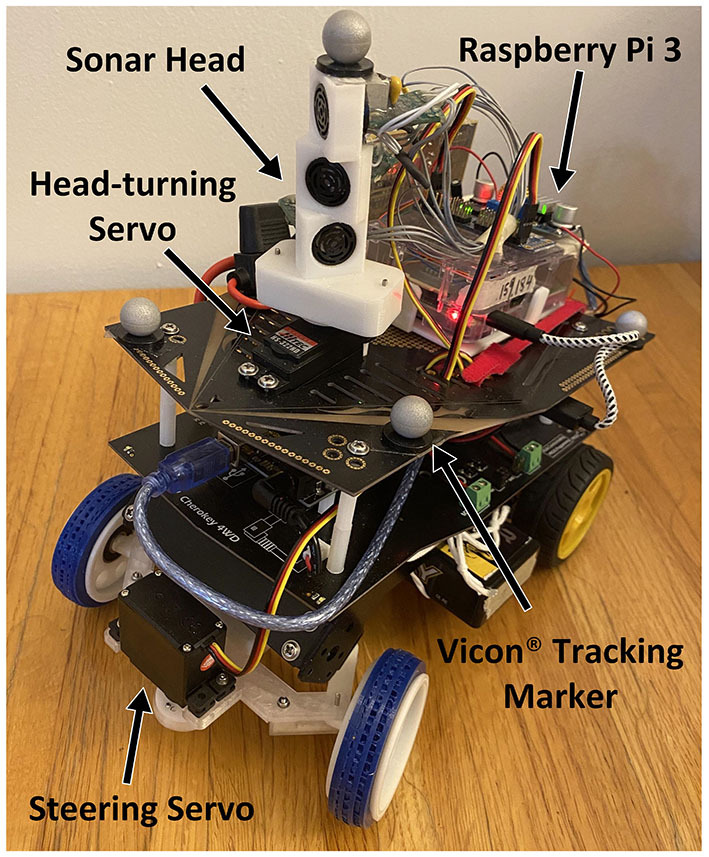
The mobile robot on which the analog model is implemented. The robot consists of a sonar head with three sonar transducers mounted on a servo motor, a Raspberry Pi 3 running the analog model, an Arduino Uno board controlling the rear wheel DC motors, a servo motor controlling the front wheels with an Ackermann steering geometry, an Adafruit 16-channel servo driver, and a lithium polymer (LiPo) battery. The sonar transducers are custom-modified MaxBotix sonar transducers (piezo) that act as both a speaker and a microphone. They resonate at 40 kHz and will only detect signals near this frequency. The transmitted beam has a half-power beamwidth of about 60°. The top transducer points 45° to the right (from the head's point of view), the middle transducer points straight forward, and the bottom transducer points 45° to the left. The trained radial basis function (RBF) network and the analog version of the Curved Openspace model are implemented in Python on a Raspberry Pi 3 mounted on the back of the robot. The Raspberry Pi allows for wireless operation via WiFi and coordinates the commands to a servo controller board (for sonar head pointing and steering) and the powered rear drive wheels. The servo motor in the front of the vehicle controls the turning rate of the vehicle using an Ackermann steering mechanism. A given steering angle thus translates to a specific turning radius of the vehicle and a lookup table is used during operation. Because of the limited resolution of the steering motor, the total number of motor actions was reduced from 33 to 15 in the robot implementation. The neural model controls the speed of the vehicle by adjusting the power delivered to the rear wheel DC motors using pulse-width modulation.

We have implemented the analog version of the Curved Openspace model on a mobile robot with a car-like steering mechanism (Figure 14). The 3-D printed sonar head (white) is mounted on a servo motor at the front of the vehicle and has three vertically stacked sonar transducers, each facing a different direction. To collect sensory data, the middle transducer sends out a sonar ping and the echoes reflected from objects are measured by all three transducers. The distances to objects are measured using the time of flight of their echoes, and the direction of each object is computed using the relative amplitudes of the sonar echo. The custom sonar transducer boards output a logarithmically-compressed envelope signal of echoes as an analog voltage. For each echo, the peak voltage on each of the three transducers is recorded by a microcontroller-based analog-to-digital converter (ADC). A radial basis function (RBF) network is then used to map the echoes to angles in the horizontal plane at 1-degree increments. The RBF network was trained using a dataset consisting of 910 echoes from 91 different angles and 10 intensities (to simulate different ranges).

The program sends out a sonar ping from the middle transducer, waits for 3 ms for the outgoing pulse to die out before collecting sonar echoes and then uses the RBF network to localize the detected obstacles. The goal input is provided in the straight-forward direction initially and it can change according to the movement of the robot to guide it toward the initial goal direction. The risk values and the desirability of 15 different paths are then calculated and a winning path along with the next ping direction is executed. Finally, the program waits for the turning of the head to finish before sending out a new sonar ping and starts the cycle again. If no head movement is required, a sonar ping is sent in the current ping direction immediately. Because of the limited rotational speed of the head-turning servo, the ping frequency varies from 3 Hz when the head needs to turn from one end to another, to 12 Hz when no head movement is required.

**Figure 15:**
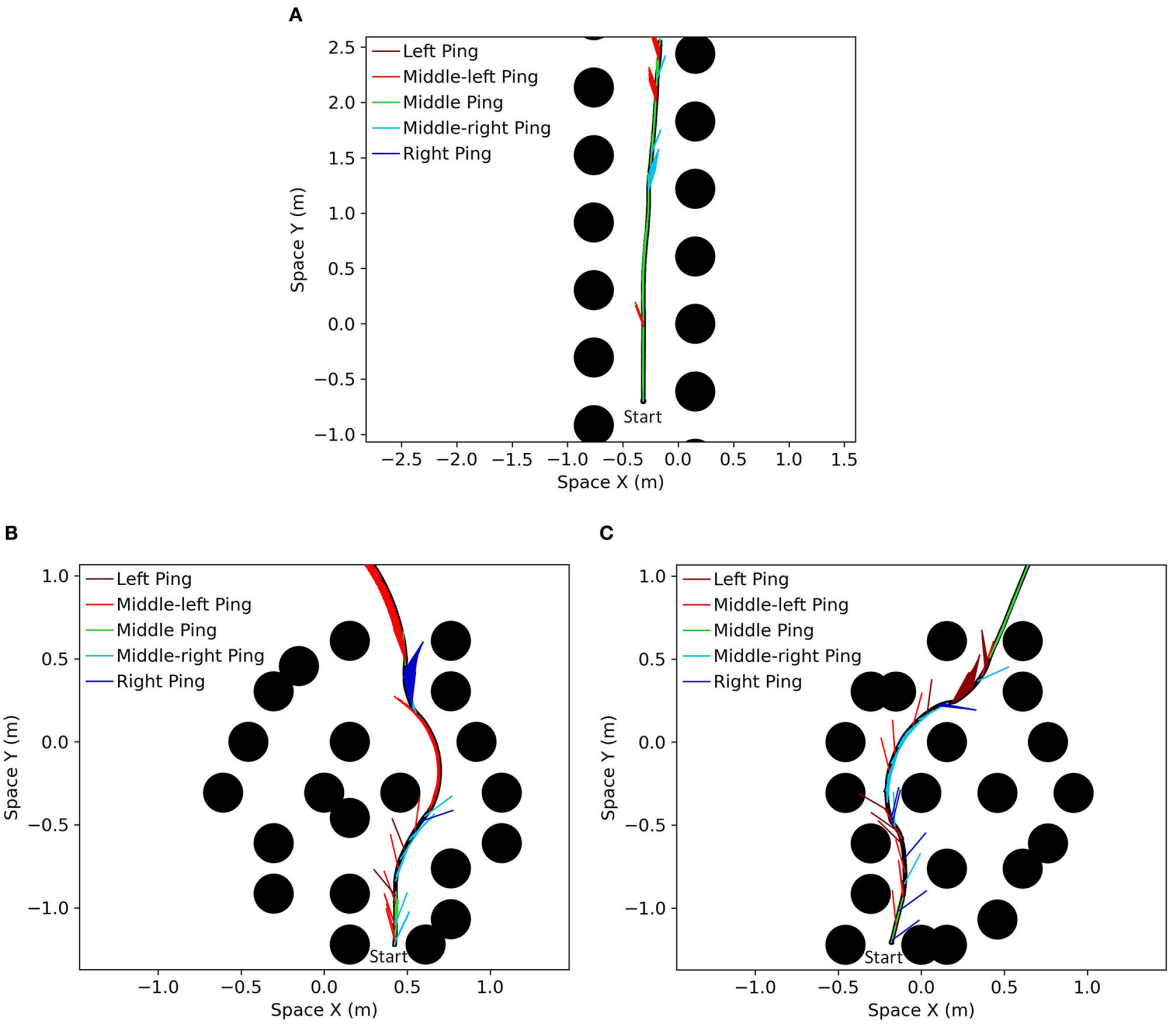
The path and ping directions of the robot during runs in three different forests. The obstacles (5 cm diameter PVC poles) are shown as black circles and the size of the circle is the size of the zone of collision (size of the robot plus the size of the PVC poles). The path the robot took is shown as a solid black line and the ping directions are shown as solid-colored lines pointing at the corresponding ping directions. **(A)** Robot running down a corridor formed by two lines of evenly spaced obstacles. The ping directions in this run were mostly down the center (green solid lines). **(B)** The robot traveling in a forest composed of 21 obstacles placed in a 1.8 × 1.8 m area. The robot explored other paths more often by pinging in different directions in more cluttered areas, whereas it mainly pinged in the direction of its current path in the more open areas. **(C)** The path of the robot in a denser forest consists of 21 obstacles in a 1.4 × 1.8 m area.

The mobile robot was tested in different forests of obstacles and its positions were recorded in 3-dimensions using a Vicon^®^ marker-based visual tracking system. The robot path and ping directions of the robot in three different example forests are shown in Figure 15. In the first configuration (Figure 15A), when the robot did not detect any obstacles near the middle path, it did not need to turn the head to other directions because the goal direction was in the middle and the middle path was the winner. As a result, most of the time the robot kept pinging down the center. The sonar pings to the side were caused by errors in the sonar RBF network that sometimes incorrectly localized side obstacles to the center. In the second configuration (Figure 15B), the area around the starting point of the robot is more cluttered, so the robot pinged around and explored different paths more often. In the second half of the run, the robot entered a more open area where it mostly pinged in the direction that it was traveling in. A similar result can be observed in a denser forest example (Figure 15C). The video recordings of the two runs shown in Figures 15B,C are provided as Supplementary Materials.

Several problems can occur in a real sonar system compared to the simulation. First, occlusion can happen when one object is directly behind another object and the sonar system cannot detect the far object accurately or at all. Another problem that can occur are multi-path echoes where the sound is bouncing between different objects, creating additional echoes with different delays that might be erroneously interpreted as different objects. During the testing of the robot, the problems mentioned above did not create significant difficulties, given our simplified environment, but we acknowledge that this might not be the case in complex 3D environments. One of the problems that we encountered, however, was the near-field *blind zone*. Following an outgoing ping, the highly resonant transducers will produce a decaying signal that continues for some time, interfering with amplitude measurements of echoes from nearby objects. Although objects can be *detected* within this signal, amplitude measurements for localization are not practical. Its range can be reduced by lowering the intensity of the outgoing ping when the robot is encountering close obstacles. Another problem that we encountered was that in certain cases, the positions of the obstacles calculated by the RBF network were inaccurate and caused the robot to steer in an undesirable direction. The incorrect motor actions were often quickly corrected in the following sensor cycle once the positions of the obstacles were accurately calculated and the incorrect memory was replaced by the accurate sensory information.

## Discussion

This paper presents an obstacle avoidance neural model that provides steering decisions to a sonar-guided “bat” to avoid static obstacles while pursuing a goal. To achieve better matching between the desired path and a path that the bat can realistically follow, the Curved Openspace model projects the sensory-based obstacle data into “motor space” before comparing motor choices. Although the idea of selecting motor actions instead of sensory directions has been proposed before (Simmons, 1996; Fox et al., 1997), they used sensors with a wide field-of-view and did not need to consider the question of how to gather information efficiently over time. Taking into account the limited field-of-view of an echolocating bat or a practical sonar system, an attentional system is proposed to control the direction of sonar pings in a time and energy efficient way where the bat will ping in the most beneficial direction to guide its motor action selection. The fact that the bat is moving while gathering and integrating information introduces problems such as the inaccuracy of old data. To alleviate the problem of inaccurate memory, we designed an evaluation memory that decays old data and a desirability function that incorporates both the information about the goal and obstacles as well as the recency of the sensory data. The presented simulations showed the effectiveness of different configurations under different situations. Although the proposed neural model is described as a model for sonar-guided creatures or vehicles, it can be driven with other sensors such as cameras or laser rangefinders.

The analog model was implemented in real-time on a car-like robot with an active sonar system. The robot was tested in multiple scenarios and the results showed that the analog model works with real sonar sensory data. During these runs, the robot would only turn its head and collect sensory data in non-traveling directions when necessary, showing the efficiency of the proposed attentional system. It is important to note that the point of the robot implementation is to demonstrate that the proposed neural model operates as expected with a real sonar and an inexpensive robot in closed-loop behavior.

A spiking neural network using spike-timing representations is also described in the paper. Instead of using a large population of neurons to represent analog signals with spike rates, we used spike-latency to represent signal values for computation. Sonar systems inherently utilize a timing code where low echo latency represents a close object and high echo latency represents a distant one. Only a small adjustment on this latency is required to translate this into the immediacy of avoidance measure. Moreover, a sonar system will receive echoes from close obstacles earlier than distant obstacles. This means that a creature implemented with the proposed spiking neural model could reduce the time to make a motor decision if it chooses to focus only on closer obstacles in a cluttered environment. With the “race-to-first-spike” WTA mechanism, an early decision can be forced by increasing the passive excitatory input to further reduce the time spent on decision-making if most of the computation happens early. The presented simulations showed that the bat running on the proposed neural network can navigate through dense forests. The slow simulation speed of the neural network, however, prevents the current simulation from running extensive performance analyses and advocates for implementation on neuromorphic VLSI hardware. Overall, the use of input spike timing to modulate the efficacy of a synaptic connection can be an effective mechanism that does not rely on increasing spike rates or modulating synaptic strength. Although the Curved Openspace model and its spiking neural model are proposed for obstacle avoidance on a 2-D plane in this paper, it would not be difficult for the neural model to operate in the 3-D world by extending the motor actions to 3-D trajectories.

## Data Availability Statement

The raw data supporting the conclusions of this article will be made available by the authors, without undue reservation.

## Author Contributions

CW contributed to designing and simulating the algorithm, testing the robot, designing and simulating the spiking neural network, and manuscript preparation. TH contributed to designing the algorithm, designing the sonar system, designing the robot, designing the spiking neural network, and manuscript preparation. All authors contributed to the article and approved the submitted version.

## Funding

This work was supported by the U.S. Office of Naval Research (N000141210339), the U.S. Air Force Office of Scientific Research (FA9550-14-1-0398, Center of Excellence), the U.S. National Science Foundation (SMA1540916, Neuromorphic Engineering Workshop), and the Maryland Industrial Partnerships Program (MIPS 2019-2021).

## Conflict of Interest

The authors declare that the research was conducted in the absence of any commercial or financial relationships that could be construed as a potential conflict of interest.

## Publisher's Note

All claims expressed in this article are solely those of the authors and do not necessarily represent those of their affiliated organizations, or those of the publisher, the editors and the reviewers. Any product that may be evaluated in this article, or claim that may be made by its manufacturer, is not guaranteed or endorsed by the publisher.
